# Identification of QTLs affecting post-anthesis heat stress responses in European bread wheat

**DOI:** 10.1007/s00122-021-04008-5

**Published:** 2022-01-05

**Authors:** Gaëtan Touzy, Stéphane Lafarge, Elise Redondo, Vincent Lievin, Xavier Decoopman, Jacques Le Gouis, Sébastien Praud

**Affiliations:** 1 Arvalis-Institut du Végétal, Biopole Clermont Limagne, 63360 Saint-Beauzire, France; 2 Centre de Recherche de Chappes, Route d’Ennezat CS90216, 63720 Chappes, France; 3 UMR 1095 GDEC, INRAE, Université Clermont Auvergne, Clermont-Ferrand, France

## Abstract

**Key message:**

The response of a large panel of European elite wheat varieties to post-anthesis heat stress is influenced by 17 QTL linked to grain weight or the stay-green phenotype.

**Abstract:**

Heat stress is a critical abiotic stress for winter bread wheat (*Triticum aestivum* L.) especially at the flowering and grain filling stages, limiting its growth and productivity in Europe and elsewhere. The breeding of new high-yield and stress-tolerant wheat varieties requires improved understanding of the physiological and genetic bases of heat tolerance. To identify genomic areas associated with plant and grain characteristics under heat stress, a panel of elite European wheat varieties (*N* = 199) was evaluated under controlled conditions in 2016 and 2017. A split-plot design was used to test the effects of high temperature for ten days after flowering. Flowering time, leaf chlorophyll content, the number of productive spikes, grain number, grain weight and grain size were measured, and the senescence process was modeled. Using genotyping data from a 280 K SNP chip, a genome-wide association study was carried out to test the main effect of each SNP and the effect of SNP × treatment interaction. Genotype × treatment interactions were mainly observed for grain traits measured on the main shoots and tillers. We identified 10 QTLs associated with the main effect of at least one trait and seven QTLs associated with the response to post-anthesis heat stress. Of these, two main QTLs associated with the heat tolerance of thousand-kernel weight were identified on chromosomes 4B and 6B. These QTLs will be useful for breeders to improve grain yield in environments where terminal heat stress is likely to occur.

**Supplementary Information:**

The online version contains supplementary material available at 10.1007/s00122-021-04008-5.

## Introduction

As one of the most important crops in the world, wheat (*Triticum aestivum* L.) has seen a steady growth in yields since the late 1960s in many areas (Calderini and Slafer 1998). However, a reversal of this trend has been observed in several countries over the last two decades (e.g., Peltonen-Sainio et al. 2009; Brisson et al. 2010; Rife et al. 2019). For example, lower yields in France have mainly been explained by climate variability causing drought stress during booting and high temperatures during grain filling (Brisson et al. 2010). It has been estimated on a world scale that climatic variations such as precipitation and heat stress may have led to a 5.5% reduction in grain yield since 1980, which corresponds to a loss of 35 M tonnes, equivalent to the annual wheat harvest of a country like France (Lobell et al. 2011). By the end of the twenty-first century, temperatures are expected to increase across most regions in the world, and on average by 0.3–1.7 °C in the lowest greenhouse gas emission scenario, and 2.6–4.8 °C in the highest one (Stocker et al. 2013). Combining climate predictions with a wheat simulation model, Semenov and Shewry (2011) showed that not drought, but the frequency and magnitude of heat stress around flowering time and during grain filling could significantly impact wheat yield, especially for wheat varieties that have not been specifically selected for their tolerance to heat stress, such as those commonly grown in Europe.

Heat stress scenarios can be characterized according to (1) the timing or the stage at which the plant is affected by stress, (2) the duration of stress, and (3) the intensity, i.e., the extremes of temperature to which the plant is exposed. Two kinds of stress scenarios are frequently associated with yield loss. “Heat shocks” are short periods (a couple of hours) of stress at very high temperatures (> 40 °C), and “chronic heat stress” is when average daily temperatures are higher than the optimum over a longer period. Crop responses to high temperature vary widely as they depend on the stress scenario, the germplasm (e.g., European wheat does not react in the same way as stress-adapted Australian wheat) and the experimental conditions (e.g., controlled or field trials). Porter and Gawith (1999) reviewed more than 65 papers describing the response of wheat to extreme temperatures and they determined that 20.7 ($\pm\,$ 1.4) °C was the optimal temperature at the grain filling stage, while 35.4 ($\pm\,$ 2.0) °C was the maximum temperature above which growth stops. To evaluate the impact of heat stress on plants, numerous studies have related plant behavior to the number of days during which the maximum temperature exceeded 25 °C at different growth stages (e.g., Sofield et al. 1977; Hunt et al. 1991; Gate 1995). Gate et al. (2010) applied this threshold to a multi-environment trial network in France and showed that for each day with a maximum temperature higher than 25 °C between heading and heading + 750 degree days (°Cd), the yield loss may be as much as 0.14 t ha^−1^.

Heat stress impacts plant development and yield through numerous mechanisms. Firstly, higher temperatures during the crop cycle accelerate some biological processes (Johnson and Thornley 1985) such that phenological stages occur earlier (Porter and Gawith 1999). While earlier completion of some growth stages could be advantageous, wheat in Europe is still impacted by heat stress events during grain filling (Gate 2007; Gate et al. 2008). There are three categories of negative physiological impacts as reviewed by Cossani and Reynolds (2012). (1) Light interception is diminished with a reduction of the leaf area index and green area duration. E.g., Xu et al. (1995) showed that a reduction of leaf chlorophyll content during a post-anthesis heat stress led to earlier senescence. (2) Radiation use efficiency (RUE) is lessened due to altered protein structure, reducing the intensity of photosynthesis, decreasing CO_2_ solubility and increasing photorespiration. (3) The partitioning of total assimilates is modified. If the stress occurs around the time of anthesis, the number of grains per spike may be impacted because of pollen sterility or grain abortion (Tashiro and Wardlaw 1990). If the stress occurs after flowering, the remobilization of water-soluble carbohydrates and starch synthesis could be impacted with consequences on grain weight (Stone and Nicolas 1994; Wheeler et al. 1996; Calderini et al. 1999). High temperature during grain cell division might also reduce the final grain size (Nicolas et al. 1984; Commuri and Jones 2001; Barnabás et al. 2007).

Senescence is a natural process during which chlorophyll is catabolized and nutrients are remobilized from the source to sink organs of the plant (Taiz and Zeiger 2002). In monocarpic species, plants which senesce later and/or more slowly than a standard reference are described as “staying green.” Stay-green plants may be able to maintain photosynthesis during grain filling (Thomas and Howarth 2000). The stay-green phenomenon, when quantified, is an indicator of tolerance under heat and drought stresses (Olivares-Villegas et al. 2007; Pinto et al. 2010; Bouffier 2014). Different techniques have been used previously to monitor senescence and extract the main parameters (e.g., start, duration, rate and end) defining the stay-green trait, such as visual scoring (Lim et al. 2007), measurement of the chlorophyll content with a SPAD meter (Borrell et al. 2000; Christopher et al. 2008) and an estimation of the normalized difference vegetative index (e.g., Lopes and Reynolds 2012; Bouffier 2014; Christopher et al. 2016).

Several genetic studies have been conducted in heat stress conditions to identify quantitative trait loci (QTL) associated with pertinent agronomical and physiological traits, reviewed by Bouffier (2014), Farooq et al. (2014), and Tricker et al. (2018). Many of these studies were performed with bi-parental spring wheat populations growing in arid environments. However, evaluating the impact of heat stress alone, without simultaneous drought stress, is challenging in field trial situations. Field studies for QTL detection by CIMMYT in Mexico (Pinto et al. 2010, 2016; Bouffier et al. 2015) circumvented this difficulty by conducting experiments with an optimal and a late sowing date while ensuring both sets of plants were well irrigated. In effect, the delayed sowing exposed plants to terminal heat stress due to high summer temperatures, but they did not suffer from any drought stress. Performing field trials to screen varieties for heat tolerance is more complicated in Europe than at the CIMMYT sites. For instance, late sowing may increase the likelihood of heat stress, but it is not certain to occur each year in Europe’s temperate climate. Moreover, testing winter wheat panels that are composed of both photoperiod-sensitive and photoperiod-insensitive genotypes may bias the results. Vernalization and photoperiod requirements may cause varieties to flower at different times, such that escape and tolerance could easily be confused under heat stress. Most studies quantifying the impact of heat stress alone have indeed used controlled environments (Stone and Nicolas 1994; Gibson and Paulsen 1999; Spiertz et al. 2006; Shirdelmoghanloo et al. 2016; Telfer et al. 2021).

The development of new improved wheat varieties would be greatly facilitated if the genetic bases of heat tolerance were deciphered. The objective of this study was to quantify the effects of high temperatures during grain filling on elite European winter wheats. Seeking QTL from European germplasm for potential introgression through a European breeding program minimizes the risk of introducing negative QTL as the genetic background is closer. Our study was conducted in controlled conditions, and each plant was exposed to a heat treatment after it had flowered without any acclimation period. Flowering time, leaf chlorophyll content, the number of productive spikes, grain number, grain weight and grain size were measured. A GWAS was then performed to identify genomic areas associated with these traits and their response to heat treatment.

## Materials and methods

### Plant material and growth conditions

A total of 199 elite European winter wheat varieties, registered in Europe from 1974 to 2010, were assembled in a diversity panel (Online Resource 1). These varieties were evaluated in the greenhouse during the spring in 2016 and 2017 at the Biogemma site in Chappes, France.

Plants were sown on 22 February 2016 and 2 March 2017 in 1 cm-deep trays containing commercial potting substrate TS 3 from Klassman (Bourgoin Jallieu, France). The seedlings were raised in the greenhouse maintained at 21 °C daytime maximum and 15 °C night-time minimum. After two weeks, seedlings were vernalized for 49 to 56 days at 8 °C with an 8-h photoperiod. Then plants were transplanted into individual 1L pots (12 cm long and 11 cm wide). The rooting medium in the pots was commercial “Fleurissement 384” from Klassman, plus a controlled-release fertilizer “Nutricote type 70” from Fertil (Boulogne Billancourt, France), with 13% N, 13% P_2_O_5_ and 13% K_2_O at 0.48 g per pot and with minor nutrients added before transplanting. Pots were watered daily and kept in trays containing water about 1 cm deep throughout the experiment to avoid water stress. Biocontrol methods and chemical pesticides were used to control diseases and pests. Air temperature and relative humidity were continuously monitored throughout the experiment at 15-min intervals using a temperature and humidity probe. Daytime maximum temperature of 21 °C was held for 12 h from 08:00 to 20:00. Similarly, night-time minimum temperature of 15 °C was held for 8 h from 22:00 to 06:00. The transition period between the daytime maximum and night-time minimum temperature was 2 h. Relative humidity was held at 60%. The photoperiod was 16 h and sodium-vapor lamps (adjusted to be 100 cm away from the plant canopy) provided a photosynthetic complement if natural luminosity was less than 90 W m^−2^.

### Split-plot design

A split-plot design was used. The first effect (whole-plot) was temperature regime, either non-stress (NS) or stress (S). The second effect (sub-plot) was the different varieties from the panel. Each treatment × variety combination was replicated three times. Fifteen tables (100 × 200 cm) were set out in a 3 × 5 matrix (Online Resource 2), and 120 pots were arranged in an 8 × 15 matrix on each table, which resulted in a density of 60 plants m^−2^. Spring wheat varieties were positioned all around the tables to minimize border effects. To compensate for the known temperature and luminosity gradients in the greenhouse, the three replicates were arranged in an East/West direction, i.e., replicate 1 on Tables 1, 4, 7, 10 and 13; replicate 2 on Tables 2, 5, 8, 11 and 14; and replicate 3 on Tables 3, 6, 9, 12 and 15. Each treatment occupied two and a half tables, e.g., for replicate 1, Tables 1, 4 and half of Table 7 were assigned to one treatment, while Tables 10, 13 and the other half of Table 7 were assigned to another treatment (Online Resource 2). The tables were split into two, defining five sub-blocks per whole-plot, where three replicate checks were placed at random (checks were the registered lines Bermude, Boregar and Premio). The relative location of temperature treatments was switched between the 2016 and 2017 experiments and different randomizations were drawn up for genotypes and check locations.

Forty thermo-buttons from the Proges Plus company (Willems, France) were used, two per table, to monitor temperature every half hour during the growth cycle. The *krige* function from the package “gstat” (Pebesma 2004) was used to perform an ordinary kriging operation and assign a mean temperature value for each pot.

### Heat treatment

Three days after anthesis (DAA), plants in the S temperature regime were put into another greenhouse at a temperature of 29 °C day/23 °C night but with the same photoperiod for ten days. The plants with the same flowering date were arranged on the same table so relative positions during this time were different from the original plot. However, after the heat treatment, the plants were returned to their former location.

The quality of temperature control was assessed by thermo-buttons. The average daytime temperatures (between 07:00 and 21:00) and night-time temperatures (between 21:00 and 07:00) in the 2016 experiment were, respectively, 30.4 $\pm\,$ 2.9 °C and 24.4 $\pm\,$ 2.6 °C for S and 20.0 °C$\pm\,$ 2.6 and 16.4 °C$\pm\,$ 2.9 for NS, and in the 2017 experiment were 29.0 $\pm\,$ 3.0 °C and 23.8 $\pm\,$ 3.5 °C for S and 21.7° C $\pm\,$ 1.7 and 17.6 $\pm\,$ 2.3 °C for NS. Relative humidity during the day and night was 49.2 $\pm\,$ 9.2% and 61.0 $\pm\,$ 8.4% for S treatment and 74.0 $\pm\,$ 10.3% and 80.2 $\pm\,$ 8.3% for NS treatment in 2016; and was 60.0 $\pm\,$ 9.6% and 70.3 $\pm\,$ 9.0% for S treatment and 77.1 $\pm\,$ 8.0% and 82.8 $\pm\,$ 6.3% for NS treatment in 2017. Vapor pressure deficit (VPD) was calculated from temperature and humidity values. VPD during the day and night, respectively, was 2.13 $\pm\,$ 0.38 kPa and 1.86 $\pm\,$ 0.23 kPa for the S treatment and 1.71 $\pm\,$ 0.21 kPa and 1.48 $\pm\,$ 0.15 kPa for the NS treatment in 2016, and 2.40 $\pm\,$ 0.37 kPa and 2.07 $\pm\,$ 0.32 kPa for the S treatment and 1.99 $\pm\,$ 0.16 kPa and 1.67 $\pm\,$ 0.16 kPa for the NS treatment in 2017.

### Measurement of yield components

For each pot, flowering time (D.Z65) corresponding to Zadoks stage 65 (Zadoks et al. 1974) was scored daily on the main shoot. At the end of the experiments, the number of fertile spikes per plant (SPP) was counted, then using a small plot harvester grains were harvested separately from the main shoot and the other shoots. Grains were dried for one week at 35 °C to ensure a homogenous moisture content before threshing. Using a MARVIN digital seed analyzer (MARViTECH, Wittenburg, Germany), the number, width, length and area of dry grains were determined for grain from both the main shoot and the tillers. The grains were weighed (GW), and the thousand kernel weights (TKW) were calculated.

### Measurement of chlorophyll content and estimation of senescence traits

A Dualex Scientific™ sensor (ForceA, Orsay, France) was used to measure chlorophyll (CHL) content (µg cm^−3^) from light transmission as follows: $CHL = \frac{Near\,infrared\,transmission-Red\,transmission}{Red\,transmission}$. CHL was measured weekly, a minimum of five different dates for each plant from flowering time until maturity. Measurements were taken at five different points along the flag leaf blade.

Senescence was modeled for each plant using the following logistic function:

1 $$CHL = D + \frac{K}{{1 + a \times \exp^{{\left( {r \times t} \right)}} }}$$

where *CHL* is a vector of the chlorophyll content (µg cm^−3^) from the flowering date to maturity, *D* is the final CHL of the dead plant, *K* corresponds to the difference in CHL between the maximum and final values, *a* and *r* are indicators of the senescence rate, and *t* is the thermal time from plant anthesis. To enable proper data fits, an extra data point corresponding to the CHL value of the fully senesced plant was added 200 °C day^−1^ after the last measurement. Fitted logistic functions were obtained using the *nls* function (Bates and Chambers 1992) from the *stat* package (R Development Core Team 2011). A first run was performed on raw data to identify outliers. To do this, the residuals distribution was compared to a normal distribution and outliers were identified using method I of the *getOutliers* function (Van Der Loo 2010) from the *extremevalues* package (R Development Core Team, 2011). A second run was then performed without the outliers to estimate the different parameters of the logistic model. Pseudo R^2^ were calculated to evaluate the strength of fitted models according to Efron's (1978) formula:

2 $${R}^{2}=1- \frac{\sum_{i=1}^{N}{({CHL}_{i}^{2}-\widehat{{CHL}_{i}})}^{2}}{\sum_{i=1}^{N}\left[{({CHL}_{i}^{2}-\overline{CHL })}^{2}\right]}$$

where *N* is the number of observations in the model, *CHL* is a vector of the chlorophyll content measurements (µg cm^−3^) from flowering to maturity,$\overline{CHL }$ is the mean of the CHL values, and $\widehat{CHL}$ is the value predicted by the model.

**Fig. 1:**
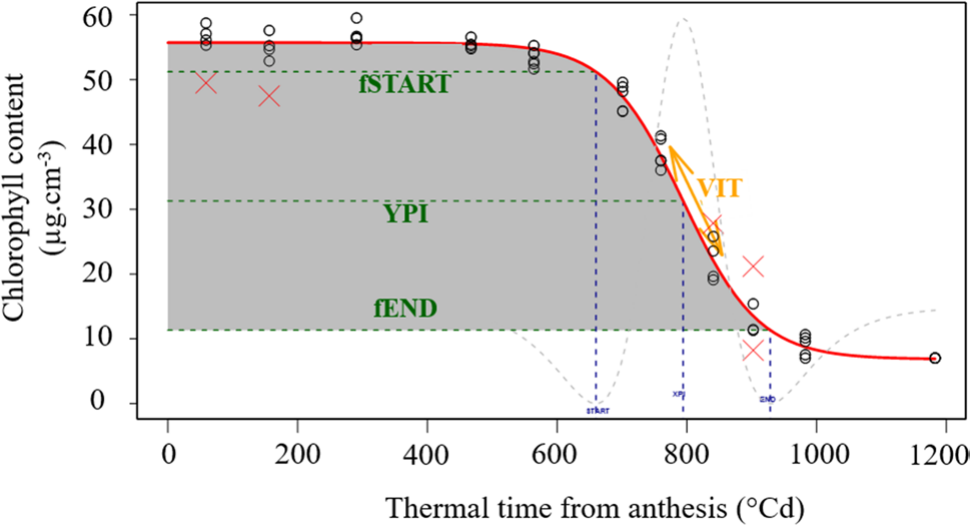
Example of the fitted logistic curve for modeling senescence and estimating stay-green traits. The red curve is an example of the fitted logistic curve for plant 455 in the 2016 experiment; the black open dots represent the raw data; the red crosses represent outliers; green and blue dashed lines are, respectively, the *y*-axis and *x*-axis coordinates of the start (START), the point of inflection (PI) and the end (END) of senescence; the dashed gray curve is the third derivative of the function which at its minima defines the start and end of senescence and at its maximum defines the *x*-axis of the point of inflection; the orange arrow indicates the maximal senescence rate; and the gray shading represents the area under the senescence curve (AUC)

The logistic function in Eq. 1 provided a close fit to the experimental data (e.g., Fig. 1). The model converged on more than 95% of the plants in both experiments. A second data cleaning operation was performed by hand to ensure that the adjustment of the kinetics corresponds correctly to the senescence process; this removal of outliers was based on senescence parameters distributions, visual curve fitness and *R*^2^. Overall, it was not possible to satisfactorily model senescence data for 199 plants (7.8%). The mean of R^2^ for the remaining plants was 0.94 ($\pm\,$ 0.05) and 0.98 ($\pm\,$ 0.02) for the 2016 and 2017 experiments, respectively. All the senescence parameters presented characteristics of normal distribution except for *a*.

Additional senescence traits were estimated using the properties of the logistic function (Fig. 1). The *x*-axis and *y*-axis of the point of inflection were obtained as $XPI= \frac{\left|\mathrm{ln}(a)\right|}{r}$ and $YPI= \frac{K}{2}$. The maximal rate of senescence was obtained with the first derivative as VIT = $f^{\prime}\left( {XPI} \right)$. The start (START) and end (END) of senescence were determined as the thermal time when the second derivative of the function attained its minimums. The chlorophyll content at these two points was noted as fSTART and fEND. We calculated the area under the senescence curve from anthesis until the completion of senescence (AUCK). It was calculated as the integral from anthesis to the end of senescence for the *x*-axis, and from K to 0 for the *y*-axis. AUCK is related to the start, the speed of senescence and the CHL maximum. We also calculated the area under the curve without the CHL maximal, named AUC. AUC is represented in Fig. 1 as the gray area, and can be considered to express the stay-green trait as it combines both the start and rate of senescence.

### Statistical analysis

The same experimental designs were used in both experiments (2016 and 2017), so we used the same baseline model to estimate genotype × treatment interaction (G × T). This model was specific to the split-plot design of our study and was adapted to a multi-year analysis and SNP by treatment interaction. The models were fitted using a mixed model written in R using the *ASReml-R* package (Butler et al. 2009).

The baseline model for the G × T estimate in one experiment was written as follows:

3 $${Y}_{ijkl}=\upmu + {R}_{j}+{T}_{k}+{T}_{k}\times {R}_{j}+{{B}_{l}(R}_{j}{T}_{k})+{G}_{i}+{{G}_{i}\times T}_{k}+{\varepsilon }_{ijkl}$$

where ${Y}_{ijkl}$ is the phenotypic value of genotype *i* (*i* = 1:199) in the sub-block *l* of the treatment *k* of the replicate *j*, *µ* is the general mean, ${R}_{j}$ is the fixed effect of the replicate *j* (*j* = 1:3), ${T}_{k}$ is the fixed effect of treatment *k* with *k* = 1 for non-stress treatment (NS) and *k* = 2 for stress treatment (S), ${T}_{k}\times {R}_{j}$ is the fixed effect of treatment *k* within replicate *j*, ${{B}_{l}(R}_{j}{T}_{k})$~ N(0, ${\sigma }_{l}^{2}$) is the random effect of the incomplete sub-block *l* (*l* = 1:5) within treatment *k* of replicate *j*, G_i_ ~ N(0, ${\sigma }_{g}^{2}$ K) is the random genotype effect where K is the genomic relationship matrix calculated using the formula from VanRaden (2008) (see below), ${{G}_{i}\times T}_{k}$ is the random genotype × treatment interaction effect with ${{G}_{i}\times T}_{k}$~ N(0, ${\sigma }_{g.t}^{2}. K\circ {I}_{nT}$), where $K\circ {I}_{nT}$ is the Hadamar product between the K matrix and the ${I}_{nT}$, identity matrix and ${\varepsilon }_{ijkl}$ is the residual error ε ~ N(0, ${\sigma }_{\varepsilon }^{2}$).

The random polygenic effect (K) was modeled with a kinship matrix as a variance/covariance matrix. Pairwise kinship coefficients were computed according to the first method described by VanRaden (2008), with a subset of 27,680 SNP obtained by removing highly correlated SNP. In brief, we computed *r*^2^ for each pair of SNP located on the same chromosome. A dendrogram was built with this matrix of *r*^2^ and one SNP was randomly selected from each cluster with a distance < 0.1 (where distance = 1−*r*^2^).

Broad sense heritability (*H*^2^) was estimated as follows:

4 $$H^{2} = \frac{{\sigma _{g}^{2} }}{{\sigma _{g}^{2} + \frac{{\sigma _{{g.t}}^{2} }}{t} + \frac{{\sigma _{\varepsilon }^{2} }}{{tn}}}}$$

where ${\sigma }_{g}^{2}$, ${\sigma }_{g.t}^{2}$ and ${\sigma }_{\varepsilon }^{2}$ are genotype, genotype by treatment interaction and residual variance, and *t* and *n* are the number of treatments and replicates.

For a combined analysis across the two years, the baseline model was enhanced with a year effect to give:

5 $${Y}_{ijklm}=\upmu +{A}_{m}+{T}_{k}+{T}_{k}\times {A}_{m}+{{R}_{j}(A}_{m})+{T}_{k}({A}_{m}{R}_{j})+{{B}_{l}({A}_{m}R}_{j}{T}_{k})+{G}_{i}+{{G}_{i}\times T}_{k}+{G}_{i}\times {A}_{m}+{\varepsilon }_{ijklm}$$

where the new terms ${A}_{m}$ and ${G}_{i}\times {A}_{m}$~ N (0, ${\sigma }_{g.a}^{2}. K\circ {I}_{nA}$) are the effects of the *m*th year and of its interaction with genotype, respectively. In this model, we must stress that part of the ${{G}_{i}\times T}_{k}\times {A}_{m}$ interaction was included in the model residual, resulting in an underestimation of the specific influence of ${{G}_{i}\times T}_{k}$ and ${G}_{i}\times {A}_{m}$.

Broad sense heritability (*H*^2^) was estimated as follows:

6 $$H = \frac{{\sigma _{g}^{2} }}{{\sigma _{g}^{2} + \frac{{\sigma _{g.t}^{2} }}{t} + \frac{{\sigma _{{g.a}}^{2} }}{a} + \frac{{\sigma _{\varepsilon }^{2} }}{{atn}}}}$$

where ${\sigma }_{g}^{2}$, ${\sigma }_{g.t}^{2}$ and ${\sigma }_{\varepsilon }^{2}$ are genotype, genotype by treatment interaction and residual variance, respectively, and *t*, *a* and *n* are the number of years, treatments and replicates.

To test the stress impact and relationship between traits the genomic best linear unbiased predictions (gBLUP) for each genotype and treatment were extracted from Eqs. 3 or 5 as follows:

7 $${\overline{GGT} }_{ik}= \widehat{\mu }+\widehat{{G}_{i}}+\widehat{{{G}_{i}\times T}_{k}}$$

Based on the ${\overline{GGT} }_{ik}$ the relationships between traits were estimated with the Pearson’s correlation and principal component analysis (PCA).

The stress index was calculated as follows:

8 $$SI=100\times {(\overline{GGT} }_{.2}-{\overline{GGT} }_{.1})/{\overline{GGT} }_{.2}$$

where ${\overline{GGT} }_{.1}$ is the average of gBLUP in stress treatment (S) and ${\overline{GGT} }_{.2}$ is the average of gBLUP in no-stress treatment (NS). This formula was calculated for each genotype to give the stress intensity at genotype level, and based on this index the correlation between traits was also calculated.

Finally, the baseline model was adapted to multi-year GWAS split-plot analysis as follows:

9 $$Y_{ijklm} = \mu + A_{m} + T_{k} + T_{k} \left( {A_{m} } \right) + \alpha_{i} + \alpha_{i} \times T_{k} + A_{m} \left( {R_{j} } \right) + T_{k} \left( {A_{m} R_{j} } \right) + B_{l} (A_{m} R_{j} T_{k} ) + G_{i}^{^{\prime}} + G_{i}^{^{\prime}} \times T_{k} + G_{i}^{^{\prime}} \times A_{m} + \varepsilon_{ijklm}$$

where the new terms ${\mathrm{\alpha }}_{i}+ {\mathrm{\alpha }}_{i}\times {T}_{k}$ represent the allelic fixed effect of the genotype *i* at SNP α and the interaction between marker α and treatment *k*, respectively. As part of the genetic effect is captured by the SNP, the notation for the genetic background is $G_{i}^{^{\prime}}$, so it is defined in the same way as the genetic effect for Eq. 5.

All the varieties were genotyped with the Affymetrix Axiom 280 K SNP array (Rimbert et al. 2018). Only the Polymorphic High-Resolution SNP were used in this analysis. All SNP were physically mapped on the genome reference sequence RefSeq V1.0 (IWGSC et al. 2018). Heterozygous loci were considered as missing data. SNP with more than 10% missing data, and SNP with a minor allele frequency (MAF) below 5% were discarded. Missing data were imputed with Beagle 4.1 (Browning and Browning 2016). Finally, 164,198 SNP were tested with Eq. 9.

To avoid proximal contamination, SNP were tested using a K matrix computed with SNP that were not located on the same chromosome as the SNP tested (Rincent et al. 2014).

The significance of random factors was tested one by one using the likelihood ratio test (LRT) (Kendall and Stuart 1979), based on log-likelihood (Lmax) differences between the complete and the reduced model without the test factor. A Wald test was performed on the complete model for testing the SNP main effect and the SNP by treatment interaction. Following the method of Gao et al. (2009), SNP were considered to be significantly associated with a trait if the -log10(*P*-value) exceeded a threshold of 5.26. The estimation of the part of the variance explained by the SNP effect on the treatment was calculated using the R^2^ resulting from the regression of the *Gi* × *Tk* coefficients depending on these effects using Eq. 5.

QTL confidence interval boundaries from GWAS results were defined following the method described in Cormier et al. (2014). Briefly, SNP belonging to the same linkage disequilibrium (LD) cluster were defined as a group of quantitative trait nucleotides. Clustering was performed by averaging *r*^2^ distances and the tree was cut at 1 “critical LD unit” (critical LD = 0.24, Breseghello and Sorrells 2006). QTL boundaries were defined as the maximal and minimal map positions of the SNP within the quantitative trait nucleotides extended by the LD decay specific to the genomic area of the QTL.

## Results

### Characterization of growth conditions and modeling senescence

A panel of 199 elite European wheat varieties were grown in controlled greenhouse conditions in a split-plot design with or without a 10-day post-anthesis heat stress in the years 2016 and 2017. NS temperatures were regulated at 21 °C daytime maximum and 15 °C night-time minimum and S temperatures at 29 °C daytime maximum and 23 °C night-time minimum. According to multiple well-spaced thermobutton measurements, on average thermal time was 27.8 °C day^−1^ and 27.1 °C day^−1^ in the S treatment and 19 °C day^−1^ and 20.6° C day^−1^ in the NS treatment, for 2016 and 2017, respectively. VPD was also calculated from the temperature and relative humidity measurements (see "Materials and methods") and though slightly higher in 2016 than in 2017, in both years of the experiment there was a clear difference in VPD between S and NS conditions. From the regular monitoring of conditions and measurement of phenotypes of plants (including the check variety) and yield components, it was possible to analyze the effect of heat stress on the panel in the absence of drought stress, because plants were well-watered throughout.

We used some of the non-invasive physical measurements to model the wheat senescence process and thus quantify the behavior of the wheat genotypes in a biologically meaningful way. Here, we modeled senescence in terms of chlorophyll content (estimated from light transmission) and thermal time using the logistic function in Eq. 1 (see "Materials and methods"). The model provided a close fit to the experimental data, as shown for one example of one genotype in one experiment in Fig. 1. The model converged on more than 95% of the plants in both years of the experiment. The second data cleaning step were found to be essential to ensure the shape of the curves corresponded to a normal biological process, for example, the total chlorophyll content at the end of senescence could have been estimated at around 40 µg cm^−3^ or −10 µg cm^−3^, which does not reflect the real distribution for this trait. For this reason, it was not possible to model the senescence of 199 plants (7.8%). The mean of R^2^ for the remaining plants was 0.94 ($\pm\,$ 0.05) and 0.98 ($\pm\,$ 0.02) for the 2016 and 2017 experiments, respectively. Additional senescence traits were estimated using the properties of the logistic function, essentially the start, the end and the rate of senescence (Fig. 1). In practice, the stay-green trait can be quantified as AUC (the gray area represented in Fig. 1) as this measure combines the start and rate of senescence.

From the regular monitoring of conditions and comprehensive measurement of phenotypes of plants (including the check variety) and yield components, it was possible to closely model the senescence process and analyze to what extent the genotype of wheat affected the observed and modeled phenotypes (Online Resource 3).

### Phenotypic analysis and variance decomposition of heat stress in wheat

Significant genotypic effects were observed for all traits except for senescence parameters *D* and *a* in both years, according to the variance components associated with the different effects extracted from the model in Eq. 3 for 2016 and 2017 experiments (Online Resource 3). Traits with significant genotypic effects showed highly variable heritability, ranging from 0.20 for fEND in 2017 to 0.95 for D.Z65 in 2016 and 2017.

As the heat treatment was applied to each plant 3 DAA, as expected, no significant impact was observed on flowering time in the 2016 and 2017 experiments. However, a slight effect of heat stress was observed on the number of fertile spikes per plant: the stress index (SI) was 3.14% (not significant, ns) in 2016 and 4.85% (*P*-value < 0.05) in 2017. The number of grains on the main shoot was not affected as the SI was 7.15% (0.05 < *P*-value < 0.01; Table 1), and on tillers there was no effect in 2016 with an SI of 8.26% (ns) and a strong effect in 2017 with an SI of 13.92% (*P*-value < 0.01; Online Resource 3). S effects on tillers were expected because they flower later than the main shoot, during or after the heat stress.

**Table 1:** Variance components for traits measured on 199 wheat varieties grown for two years with two post-flowering temperature treatments

Variable		Units	Mean	Sd	H^2^	SI	ssY	ssT	ssYT	varG	varGT	varGY	varRes
Flowering Date	D.Z65	D	175.7	6.84	0.88	-0.06	32.17	0.31	0.57	24.29***	0.14	5.03***	9.04
Senescence Parameters	D	µg cm^−3^	5.10	1.09	0.05	1.66	118.08***	0.4	4.09	0.04	0.25**	0.25*	6.27
	K	µg cm^−3^	46.14	3.16	0.77	-0.27	15.5	2.74	40.45	7.62***	2.07E-06	1.62***	17.44
	A	µg cm^−3^	6.15E-04	4.90E-04	4.31E-03	-29.22	1.08E-04*	4.35E-06	1.99E-06	7.68E-09	1.66E-07	1.07E-07	1.95E-05
	R	µg cm^−3^ Cd^−1^	2.28E-02	4.94E-03	0.18	-1.67	1.36E-03***	4.05E-06	8.36E-05	2.16E-06	2.71E-06**	5.19E-06***	6.70E-05
Senescence traits	XPI	°Cd	657	82.54	0.81	3.75	164,647***	25,379	20,941	3778***	143	508***	6675
	YPI	µg cm^−3^	28.2	2.08	0.74	0.11	67.25***	0.03	0.46	2.09***	0.13*	0.62***	4.32
	VIT	µg cm^−3.^ °Cd^−1^	-0.26	0.06	0.33	-2.28	0.09***	8.18E-04	0.01	6.47E-04**	3.69E-04***	9.31E-04***	0.01
	START	°Cd	532	67.39	0.74	5.39	18,764	28,689*	6276	2897***	221	665***	6819
	fSTART	µg cm^−3^	47.0	3.28	0.81	-0.04	64.1**	0.03	5.3	6.58***	0.06	1.57***	9.13
	END	°Cd	781	107.61	0.74	2.62	350,664***	23,832	63,877*	4814***	226	1051***	12,938
	fEND	µg cm^−3^	9.3	1.16	0.14	0.81	121.98***	0.3	1.9	0.11	0.22**	0.27**	5.26
	AUC	µg cm^−3^°Cd	26,828	4176	0.79	3.75	244,029,819***	48,250,209	13,692,149	9,649,426***	247,761	2,040,704***	16,651,766
	AUCK	µg cm^−3^°Cd	34,148	5746	0.81	3.72	582,017,317***	54,314,087	29,880,570	15,481,554***	629,865*	2,995,929***	20,730,987
Number of Spikes	SPP	plant^−1^	4.8	1.05	0.70	4.01	2.67	2.07	1.07	0.64***	0.06*	0.21***	1.67
Main Shoot	NbGrainS		39.5	8.11	0.80	7.15	184.54^†^	190.65^†^	65.76	39.5***	4.29***	5.48***	57.16
	GW	g	1.54	0.45	0.72	28.74	0.49*	3.3***	0.19	0.06***	0.01***	0.01***	0.11
	TKW	g	39.1	7.29	0.64	23.90	166.16*	1582.09***	141.67*	12.3***	4.24***	4.39***	32.20
	Grain.Area	mm^2^	14.5	1.65	0.72	14.40	6.53*	70.3***	2.84	0.85***	0.2***	0.2***	1.53
	Grain.Width	mm	3.42	0.24	0.66	10.01	0.07	1.83***	0.16*	0.01***	4.62E-03***	4.99E-03***	0.03
	Grain.Length	mm	5.84	0.29	0.85	4.53	0.26*	1.24***	0.01	0.04***	4.54E-03***	4.49E-03***	0.04
Tillers	NbGrainS		138	35.38	0.71	11.24	162.56	8587.89*	3586.11	765.63***	111.84***	207.08***	1813.77
	GW	g	5.37	1.64	0.68	26.24	0.62	57.14***	0.36	1.14***	0.25***	0.34***	2.97
	TKW	g	38.9	6.04	0.69	17.54	27.79	676.46***	96.72*	10.9***	2.93***	3.21***	20.80
	Grain.Area	mm^2^	14.0	1.43	0.78	10.68	4.25*	30.97***	6.59*	0.85***	0.15***	0.13***	1.10
	Grain.Width	mm	3.37	0.20	0.75	6.65	0.03	0.68***	0.23**	0.01***	3.36E-03***	2.24E-03***	0.02
	Grain.Length	mm	5.71	0.29	0.86	4.16	0.19*	0.94***	0.07	0.05***	3.93E-03***	5.48E-03***	0.03

*Abbreviations* mean, mean for fitted values; Sd, standard deviation for fitted values; H^2^, broad sense heritability; SI, stress index; varT, variance explained by the treatment (T); varG, variance explained by the genotype (G); varG** × **T, variance explained by the G × T interaction; varG** × **Y, variance explained by the G × Year interaction; varRes, residual variance. Wald and LTR test: ^***^P < 0.001; ^**^P < 0.01; ^*^P < *0.05; *^*†*^*P* < *0.1, non-significant P* > *0.05*

**Fig. 2:**
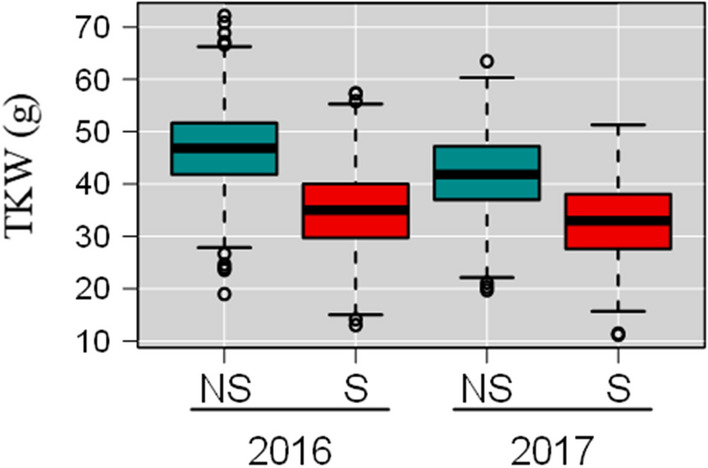
Boxplot of TKW for 199 wheat cultivars grown over two years (2016 and 2017) with two post-flowering temperature treatments (NS no stress, S stress). gBLUP values calculated from Eq. 2 were used (see "Materials and methods"). Quartiles and medians are used to construct the boxes. Whiskers extend to 1.5 times the interquartile range from the box. The means of each sample are significantly different from each other (α = 0.001) based on Tukey HSD test

Considering the senescence traits, only the *x*-axis of the point of inflection (XPI) and the end of senescence (END) in 2016 were significantly affected by the S conditions with SI of 6.89% and 6.44%, respectively. Heat stress impacted the area, length and width of grain from both the main shoot and the tillers in both years. For the TKW of the grain from the main shoot, the means were 46.77 g in 2016 and 41.96 g in 2017 for the NS treatment, but 34.88 g and 32.86 g for the S treatment (Fig. 2). S reduced the TKW of the main shoot by 25.73% in 2016 and 21.88% in 2017. The performance of the varieties and their SI are available in Online Resource 4.

Variance decomposition revealed significant G × T interactions for TKW, grain area, grain width and grain length for both the main shoot and the tillers in 2016 or 2017 (Online Resource 3). The ratio of the G × T variance on the sum of the genetic and G x T variances was the highest for TKW and grain width on the main shoot. For TKW, this ratio was 22.45% in 2016 and 22.76% in 2017. For other traits, the G × T interaction varied between the two years.

### Multi-year phenotypic analysis and variance decomposition

Variance components associated with different effects were extracted from the model (Eq. 5), combining the 2016 and 2017 experiments, and are presented in Table 1. Significant genotypic effects were observed for all traits except for senescence parameters *D*, *a* and *r*, and fEND. Traits with significant genotypic effects showed highly variable heritability ranging from 0.33 for the rate of senescence (VIT) to 0.88 for the flowering date (D.Z65).

**Fig. 3:**
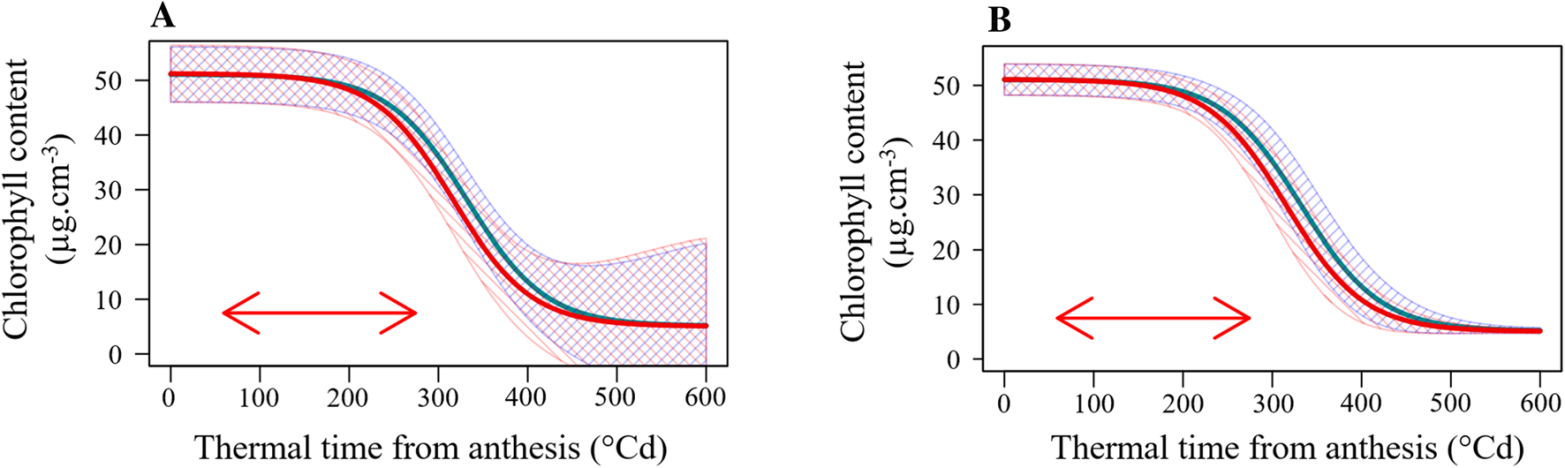
Senescence kinetics over thermal time after anthesis for 199 wheat varieties grown with two post-flowering temperature treatments. The mean (curves) $\pm\,$ standard deviation (shaded areas) of the fitted logistic senescence curve calculated from **a** raw data for both years or **b** from gBLUP parameters are shown for all genotypes in stress (red) and no stress (blue) treatments. The red double-headed arrow represents the period during which the stressed plants were exposed to heat treatment

No significant effect of treatment was observed for D.Z65, SPP and senescence parameters. Only slight effects were observed for XPI, START and AUC with SI of 3.75%, 5.39% and 3.75%, respectively. The shapes of the mean senescence curves were similar but in S conditions senescence started earlier (Fig. 3). A mildly significant stress effect was observed for the number of grains on the main shoot and on tillers, the SI being 7.15% and 11.24%, respectively. For other traits such as TKW and grain weight, area, width and length, a significant effect was observed with an SI varying from 4.16% for the length of grain from tillers to 28.74% for the weight of grain from the main shoot.

The year effect was most significant for senescence traits (Table 1), with only a weak effect on other traits. Average TKW was higher in 2016 than in 2017 (Fig. 2).

Looking at the interactions, we found a significant G × Y interaction for all traits, except for senescence parameter *a*. The genetic variance of the senescence rate (VIT) shows that this trait has the strongest G × Y interaction. The grain parameters showed significant high G × T effects.

For TKW, the variance explained by G × T and G × Y interactions was similar for the main shoot at 4.24 g^2^ and 4.39 g^2^, and slightly lower for the tillers with 2.93 g^2^ and 3.21 g^2^, respectively. This strong G × Y effect could be explained by uncontrolled environmental factors such as global radiation, which was far lower in 2016 than in 2017, even when supplemented with sodium-vapor lamps.

### An overview of senescence traits

The variance components of senescence traits are detailed in Table 1. The change in heritability and the response of senescence to the temperature treatment was reported for the whole grain filling period. Figure 3 compares the senescence curve variability based on raw data per plant and based on gBLUP of the senescence parameters. For raw data, the size of the confidence interval (CI) around the mean ($\pm\,$ one standard deviation) remained stable during senescence until around 400 °C day when the CI widened considerably (Fig. 3a). By contrast, for the gBLUP of the senescence parameters (Fig. 3b), the CI narrowed at the end of senescence. In fact, varieties are declared as a random effect in the mixed model, so if there are no significant differences among genotypes the shrinkage factor will tend toward infinity and breeding value predictions will tend to the mean (low heritability). Figure 3 shows that while there are differences between genotypes at the beginning of senescence, all the genotypes converged to values which are not discriminating. Furthermore, as the figure summarizes the distribution of senescence kinetics for all genotypes in both treatments, we can see that the heat treatment only had a slight effect on the senescence curves (the SI is at 5.39 for START and 3.75 for XPI).

### Genetics correlation

**Fig. 4:**
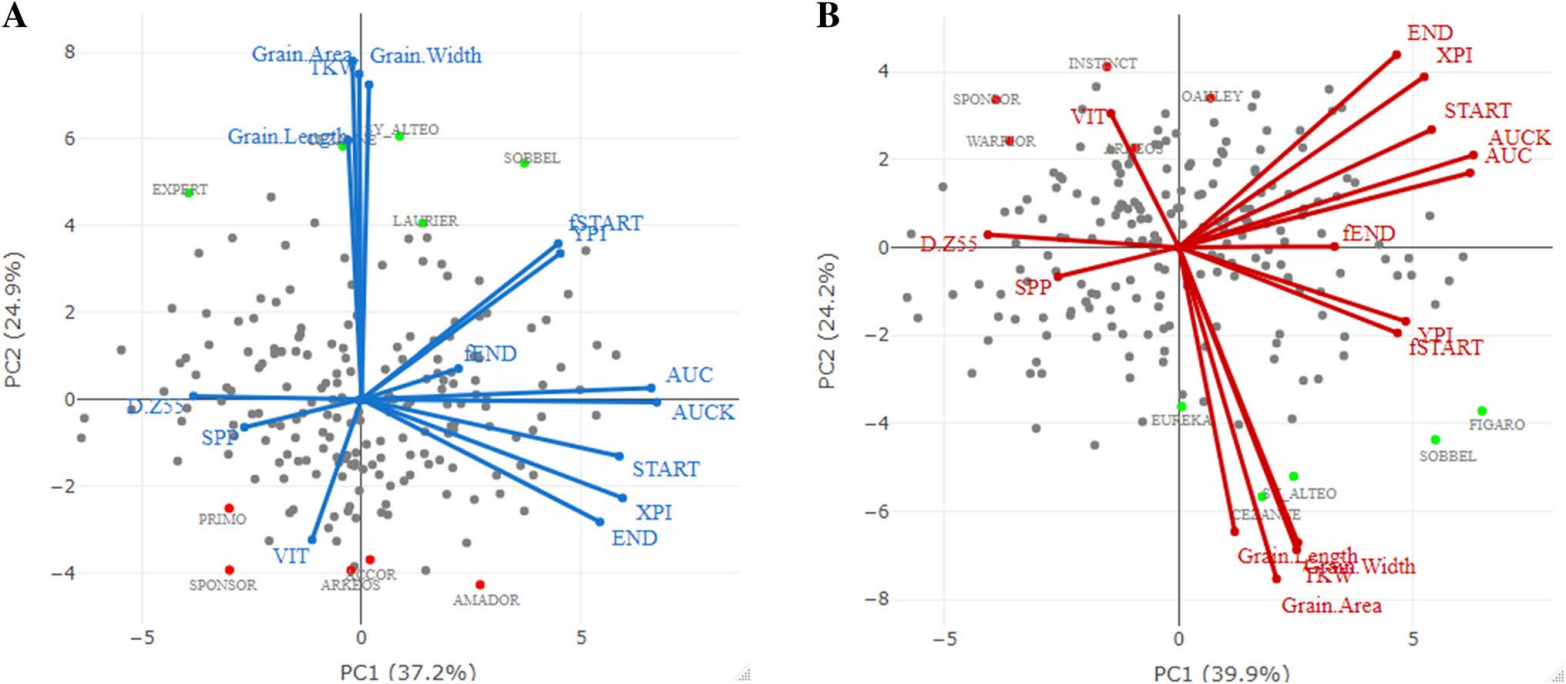
Biplots of the principal components of a subset of traits (lines) measured on 199 wheat varieties (dots) grown in **a** no stress and **b** stress post-flowering temperature treatments. The varieties with the five highest and five lowest main shoot TKW values are, respectively, plotted as green and red dots with their names given. For details of trait names see "Materials and methods"

Relationships among traits and genotypes for each heat treatment were investigated using Pearson’s correlation and biplot analysis with gBLUP estimated per treatment from Eq. 7. All correlation data are presented in Online Resource 5. Figure 4 shows the PCA of a subset of traits related to the characteristics of grain from the main shoot, senescence traits, flowering time and number of fertile spikes per plant where vector length shows the extent of variation explained by each trait. The first two axes explained up to 62% of total variability in NS (Fig. 4a) and 64% in S (Fig. 4b) treatments, respectively. The relationships between traits is similar for both treatments. Two main clusters of traits are discernible: (1) traits related to the grain such as TKW, and grain area, length and width, and (2) senescence traits. No significant correlation was detected between D.Z65 and TKW in the NS treatment (*r* = 0.08, *p* > 0.1), but a significant weak correlation was detected between these two traits in the S treatment (*r* =−0.24, *p* < 0.001). We also detected significant weak correlations (*p* < 0.05) between TKW and the respective senescence traits of YPI (*r* = 0.19), fSTART (*r* = 0.20), END (*r* =−0.16) and VIT (*r* = 0.19) in the NS treatment. In the S treatment, we still detected correlations between TKW and, respectively, YPI (*r* = 0.26), fSTART (*r* = 0.27), and VIT (*r* =−0.18) but not between TKW and END, while a positive correlation was detected with AUC (*r* = 0.19).

### Genome-wide association study

**Fig. 5:**
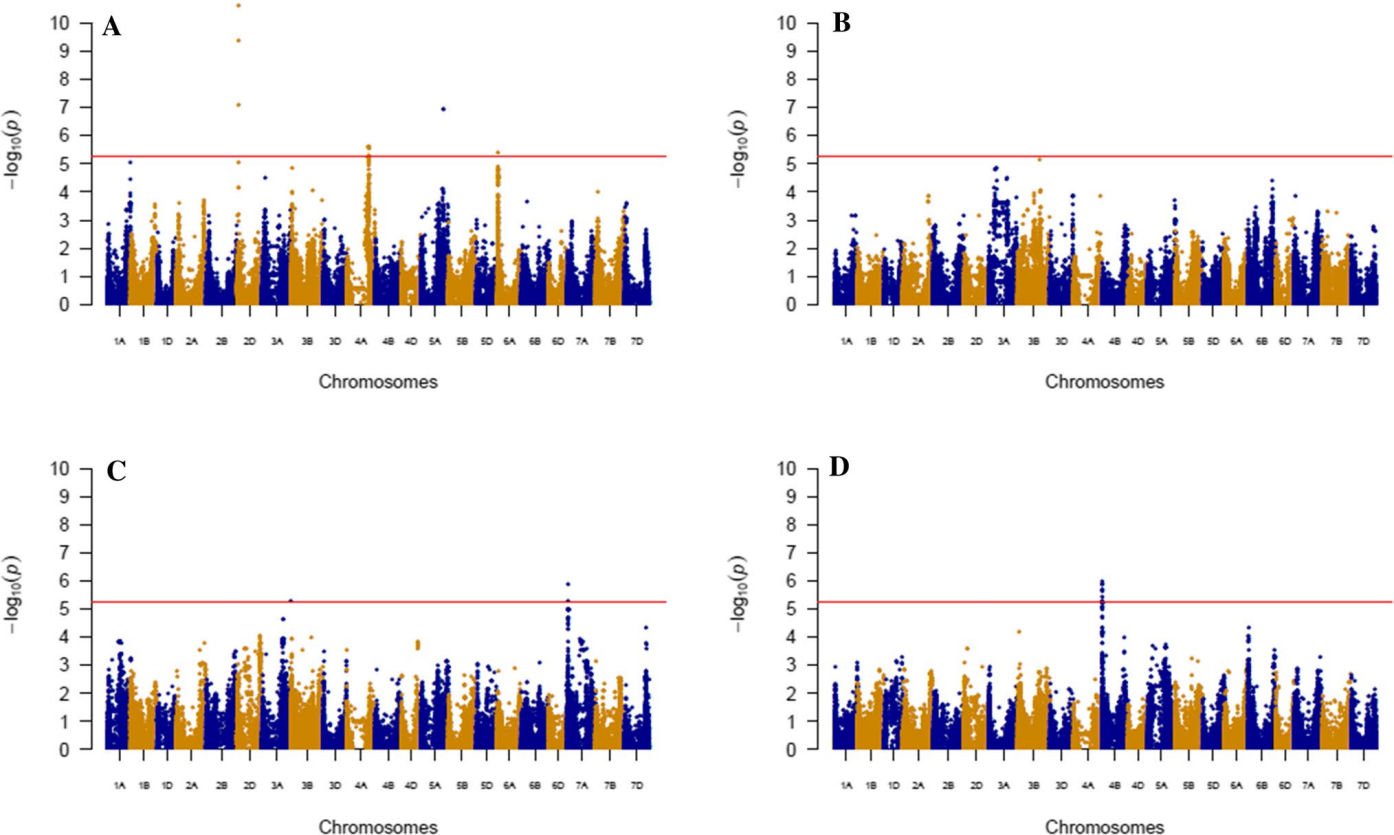
Manhattan plot of the GWAS of the SNP main effect and the SNP × treatment interaction for flowering date (D.Z65) and TKW measured on 199 wheat varieties grown in stress or non-stress post-flowering temperature treatments. **a** SNP main effect for D.Z65; **b** SNP by treatment interaction for D.Z65; **d** SNP main effect for TKW; **c** SNP by treatment interaction for TKW. The red line represents the 5.6 significant threshold calculated as described by Gao et al. (2015)

A single-locus genome-wide association study, involving a total of 164,198 SNP, was performed using raw data from both years. The main effect and the SNP × treatment interaction were tested using Eq. 9. All phenotypic traits were tested, except the senescence parameters *a* and *D*, and fEND which had not shown significant genotypic effects. Four genomic areas were significantly associated with the SNP main effect for flowering date considering the estimated 5.26 threshold for -log (*P*value) (Fig. 5a, Table 2). No SNP × treatment interaction was significantly associated with flowering date (Fig. 5b). TKW from the main shoot was only associated with an SNP main effect on chromosome 7A and to an SNP × treatment interaction effect on chromosome 4B (Fig. 5c, d, Tables 2 and 3).

**Table 2:** List of associated SNP following a GWAS conducted on 199 wheat varieties grown for two years with two post-flowering temperature treatments: SNP with no interaction effect

	Trait	Units	Chr	Position (bp)	SNP	Major Allele	Freq	SNP main effect				QTL boundary		
								LOD	Major allele effect	Effect SD	QTL	Nb. SNP	Min. (bp)	Max. (bp)
Flowering Date	D.Z65	d	4A	576,159,104	AX-89667446	A	0.64	5.62	3.98	2.35	QTL.05	13	575,485,603	581,337,826
	D.Z65	d	5A	584,428,207	AX-89390208	C	0.85	6.93	6.16	2.42	QTL.07	2	583,039,479	585,816,937
	D.Z65	d	6A	13,010,065	AX-89584501	G	0.55	5.39	3.63	2.34	QTL.08	1	10,570,285	15,449,845
	D.Z65	d	2D	34,995,194	AX-89382235	T	0.52	10.62	−5.71	2.22	QTL.04	3	32,510,457	36,442,547
Senescence	XPI	°Cd	4D	17,083,871	AX-89398511	A	0.67	5.77	−65.29	29.72	QTL.06	4	16,992,006	18,830,860
	XPI	°Cd	2D	34,995,194	AX-89382235	T	0.52	6.11	50.98	28.82	QTL.04	1	33,532,314	36,458,074
	VIT	µg cm^−3^°Cd^−1^	2A	45,486,914	AX-89761338	G	0.84	5.54	0.04	0.02	QTL.01	1	44,809,106	46,164,722
	START	°Cd	2D	34,995,194	AX-89382235	T	0.52	6.24	56.48	26.04	QTL.04	2	33,217,357	36,453,954
	END	°Cd	4D	17,083,871	AX-89398511	A	0.67	7.06	−83.51	34.52	QTL.06	16	16,826,225	23,189,173
Number of Spikes	SPP	plant^−1^	2B	25,953,023	AX-89528410	A	0.82	6.56	1.00	0.41	QTL.02	3	24,831,122	26,711,709
	SPP	plant^−1^	2B	619,396,366	AX-89729195	T	0.91	6.47	1.82	0.44	QTL.03	170	605,382,213	652,111,584
Main Shoot	NbGrain		4D	19,694,106	AX-89363486	G	0.64	6.88	7.44	3.28	QTL.06	21	16,840,726	23,189,154
	GW	g	4D	17,083,871	AX-89398511	A	0.67	9.37	0.38	0.14	QTL.06	32	16,664,691	25,447,819
	TKW	g	7A	16,603,832	AX-89332971	G	0.68	5.87	3.60	1.84	QTL.09	3	15,907,587	17,300,104
	Grain.Area	mm^2^	7A	16,603,832	AX-89332971	G	0.68	5.66	0.87	0.46	QTL.09	2	15,907,587	17,300,104
	Grain.Width	mm	7A	16,603,832	AX-89332971	G	0.68	6.13	0.12	0.06	QTL.09	3	15,907,587	17,300,104
Tillers	NbGrain		4D	19,459,578	AX-89445201	G	0.61	6.44	35.82	15.11	QTL.06	9	16,839,627	22,372,934
	GW	g	4D	17,430,704	AX-89345211	A	0.64	8.99	1.69	0.61	QTL.06	28	13,163,753	24,863,780
	TKW	g	7A	25,929,273	AX-89765935	C	0.63	5.86	3.13	1.71	QTL.10	21	25,228,927	26,941,996

*Trait abbreviations* D.Z65, flowering date; XPI and YPI, *x*-axis and *y*-axis of the inflexion point of senescence curve; VIT, rate of senescence, START, beginning of senescence; END, end of senescence; SPP, number of fertile spikes per plant; NbGrain, number of grains; GW, grain weight; TKW, thousand kernel weight; Grain.Area, area of grain; Grain.Width, width of grain. Position is relative to the reference genome Chinese Spring refSeq1.0 (IWGSC et al. 2018)

**Table 3:** List of associated SNP according to a GWAS of 199 wheat varieties grown for two years with stress or no-stress post-flowering temperature treatments: SNP with a significant interaction effect

	Trait	Units	Chr	Position (bp)	SNP	Major Allele	Freq	SNP by treatment interaction				QTL boundary		
								LOD	Effect of the major allele effect in S	Effect SD	QTL	Nb. SNP	Min. (bp)	Max. (bp)
Senescence	XPI	°Cd	7B	693,020,724	AX-89578121	C	0.91	5.71	60.40	34.72	QTL.17	2	692,252,452	693,926,665
	XPI	°Cd	1D	17,642,951	AX-89766697	A	0.62	5.38	−32.45	31.54	QTL.12	1	13,902,574	21,383,328
	YPI	µg cm^−3^	1B	631,711,683	AX-89638269	G	0.81	6.69	1.24	0.82	QTL.11	2	630,767,028	632,656,378
	AUCK		4A	328,128,331	AX-89642074	A	0.91	5.55	−3119.83	2355.64	QTL.14	36	38,974,540	472,729,606
Main Shoot	TKW	g	4B	1,841,022	AX-89364414	G	0.64	5.99	−3.51	2.15	QTL.15	11	1,339,055	2,852,831
	Grain.Area	mm^2^	4B	1,853,262	AX-89500218	A	0.63	6.42	−0.78	0.53	QTL.15	34	1,384,350	3,700,062
	Grain.Width	mm	4B	1,853,262	AX-89500218	A	0.63	5.83	−0.11	0.07	QTL.15	9	1,310,533	2,334,232
	Grain.Length	mm	4B	1,854,364	AX-89738651	T	0.64	5.49	−1.14E-01	1.10E-01	QTL.15	25	1,438,558	3,697,489
Tillers	GW	g	3B	150,697,370	AX-89468413	C	0.91	5.40	1.48	0.74	QTL.13	1	141,244,647	160,150,093
	TKW	g	6B	695,794,696	AX-89726711	A	0.58	5.76	2.53	1.96	QTL.16	7	694,970,068	696,420,120
	Grain.Area	mm^2^	6B	695,794,696	AX-89726711	A	0.58	5.85	0.58	0.51	QTL.16	8	694,970,068	696,420,120
	Grain.Length	mm	6B	695,794,696	AX-89726711	A	0.58	6.13	1.01E-01	1.10E-01	QTL.16	9	694,970,068	696,420,120

*Trait abbreviations* XPI and YPI, respectively, the *x*-axis and *y*-axis of the inflection point of the senescence curve; AUCK, total surface under the senescence curve; GW, grain weight; TKW, thousand kernel weight; Grain.Area, area of grain; Grain.Width, width of grain; Grain.Length, length of grain. Position is relative to reference genome Chinese Spring refSeq1.0 (IWGSC et al. 2018). S, heat stress treatment

**Fig. 6:**
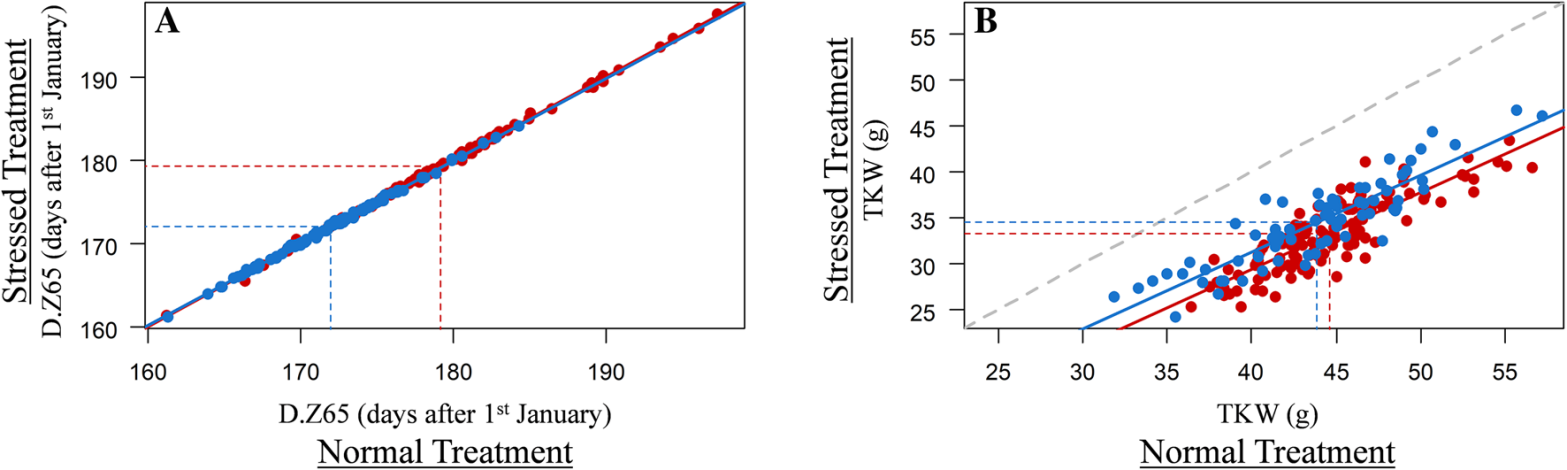
Correlation between gBLUP values for 199 wheat varieties grown in two post-flowering temperature treatments. **a** Correlation between flowering dates for stressed and non-stressed plants showing the minor allele (T) effect of QTL 2D (blue) and the effect of the other allele (red). **b** Correlation between TKW for stressed and non-stressed plants showing the minor allele (G) effect of QTL 4B (blue) and the effect of the other allele (red). Dotted lines correspond to average values and solid lines to linear regressions for the minor allele (blue) and the other allele (red)

Allelic effects for the most strongly associated SNP for flowering date (chromosome 2D) and the most strongly associated SNP for TKW (4B) are plotted in Fig. 6. First, we can see that there was no genotype dispersion around the bisecting line for flowering date (Fig. 6a), because there was no genotype × treatment interaction, whereas a large dispersion is observed for TKW (Fig. 6b). Second, the figure shows the difference between SNP alleles. For the SNP significantly associated with the main effect (Fig. 6a), genotypes are separated only along the bisecting line (*y* = *x*). However, for the SNP significantly associated with treatment interaction (Fig. 6b), genotypes are separated following the second bisecting line (*y* = -*x*).

Of the four QTLs associated with flowering date (Table 2), the strongest one is located on chromosome 2D colocalized with the photoperiod sensitivity gene *Ppd-D1* (Beales et al. 2007). This QTL also colocalized with the start of senescence and XPI traits. The effect of the minor allele had opposite effects, meaning that earlier flowering genotypes had a delayed start of senescence. Two more QTLs were associated with senescence traits, one on chromosome 2A was associated with the maximum senescence rate (VIT), and one on chromosome 4D was associated with the start and end of senescence and colocalized with the *Rht-D1* dwarfing gene (Peng et al. 1999). The latter QTL was also associated with the number of grains per spike in the main shoot and tillers, but with an opposite effect to those of the associated senescence traits in that early senescing plants had more grains and heavier spikes than later senescing ones. Lastly, one QTL localized on chromosome 7A was associated with TKW for both the main shoot and the tillers.

Seven QTLs were significantly associated with the SNP × treatment interaction effect (Table 3). Four were related to senescence traits, one located on chromosome 4B was associated with main shoot TKW and related grain traits (area, length, and width), and two located on chromosomes 3B and 6B, respectively, were associated with grain weight and TKW traits for tillers. All the effects of the major allele of the most significant SNP per QTL are presented in Table 3. For example, varieties with allele G at QTL 4B, which is the major allele with a frequency at 0.64, performed less well in S treatment than varieties with allele A. This effect depicted in Fig. 6a amounts to −3.51 ($\pm\,$ 2.15) g. None of the QTLs associated with an SNP by treatment interaction showed a significant SNP main effect.

## Discussion

This study explored the impact of post-anthesis heat stress on a European elite bread wheat panel in controlled conditions. Physiological traits such as flowering time and grain morphology were recorded and senescence was modeled. We performed a GWAS to dissect the genetic determinants of heat tolerance. We identified 10 QTLs associated with at least one trait as a main effect and seven QTLs associated with an interaction with post-anthesis heat stress.

### Experiment validation and stress impact

Before interpreting GWAS results, it is important to correctly describe the stress undergone by the plants. Post-anthesis temperature was increased daily for 10 days by on average + 8.6 °C in the day and + 5.8 °C at night. This heat stress reduced grain weight by 24% for the main shoot compared with the optimum temperature of 21 °C in the day and 17 °C at night. This result is consistent with other reports of grain weight loss under post-anthesis heat stress (Stone and Nicolas 1994; Gibson and Paulsen 1999; Spiertz et al. 2006). The TKW decrease was correlated with a reduction in grain width (SI = 4.5%) rather than in grain length (SI = 10.0%, Online Resource 6). The higher sensitivity of grain width over length has already been reported (Jamil et al. 2017).

We decided to apply the S condition three days after the main shoot flowered, so as not to impact the number of grains on the main shoot. This was indeed the case as for both years the number of grains per main shoot was not significantly different between the two temperature treatments (Table 1). The impact of heat stress was therefore focused on only one of the yield components, TKW. However, it has been shown that high pre-flowering temperatures may cause flower sterility (e.g., Farooq et al. 2011; Barber et al. 2017). Yield components develop one after the other and there are multiple compensation mechanisms at work that are liable to bias the interpretation (e.g., Slafer and Andrade 1993; Gate 1995). In our case, fewer grain per spike could have caused an increase in thousand kernel weight. Furthermore, when plants survive a long period of stress, they may set in motion various acclimation mechanisms with different genetic determinisms (Gaspar et al. 2002). Our experiment was designed solely to identify heat stress QTL specific to the early phase of the main shoot grain filling. For the same reason, we chose to analyze grains from the main shoot and from tillers separately. Heat stress may have a different impact on the main shoot and tillers, due to the naturally sequential flowering dates for tillers, up to nine days later than the main shoot (Jones et al. 2017). For example, heat stress may impact the number of grains on tillers by reducing pollen fertility (Farooq et al. 2011), while the fertilization has already occurred on the main shoot. In both our experiments (2016 and 2017), the heat stress decreased the number of grains on tillers by more than 5%, while decreasing grain weight for both the main shoot and tillers, with SI at 23.9% and 17.5%, respectively. The smaller decrease for tillers could be due either to compensation for having fewer grains and/or the shorter period of stress experienced due to later flowering.

For monocarpic species exposed to terminal heat stress, it is useful to determine whether the stress damages the photosynthetic source more than the reproductive sink. In this study, we monitored leaf chlorophyll content during grain filling and observed only a weak impact of stress on senescence traits. The significant treatment effects for senescence parameters were for the number of °Cd up to the start and inflexion point of senescence, which had SI of 5.26% and 3.75%, respectively. These observations confirm the view that high temperatures, as applied in our conditions, shorten the plant cycle and limit the amount of light intercepted (Sofield et al. 1977). Clearly, heat stress accelerates the rate of grain filling while shortening its duration (Sofield et al. 1977; Dias and Lidon 2009). It has been estimated that for every °C above the optimal growing temperature, the duration of grain filling is reduced by 2–3 days (Streck 2005). Such an impact was visualized in our results by plotting the decrease of chlorophyll content as a function of Julian days for both treatments (Online Resource 7), but when senescence dynamics was plotted as a function of degree days the difference was less marked (Fig. 3, Tables 1).

We applied heat stress just after anthesis so it is likely that the number of endosperm cells was altered with consequences on grain filling and final grain size. Indeed, wheat grain usually develops in three phases: the lag phase, filling and maturation (Gate 1995; Acevedo et al. 2002). During the lag phase, which lasts about 15 days or 250°Cd, the number of endosperm cells is fixed. While the supply of assimilates available to the grain during the first two weeks after anthesis is thought to determine the cell number (Brocklehurst 1977), once fixed, it is the number of cells that regulates the rate of accumulation of dry matter during the filling phase.

This work was carried out on a large collection of elite European winter varieties that were not specifically bred for heat stress tolerance. A large variability was observed among the grain and senescence traits measured. Specifically, a large G × T variance was observed for grain weight, meaning that genotypes responded very differently to heat stress. This information could already be useful for breeders in assessing the performance of the varieties and selecting new genitors for breeding crosses. For example, varieties such as Artdeco and Saint-Ex only lost about 10% of their TKW while others lost more than 35% (Online Resource 4). The large variability observed was associated with moderate to high heritability (Table 2) meaning that this experiment was suitable for detecting QTL linked with these traits.

### QTL associated with measured traits

We detected 10 QTLs associated with at least one trait main effect (Table 2). Three QTLs were associated with senescence traits. QTL 4D is co-located with the *Rht-D1* dwarfing gene. *Rht* genes have many effects on wheat development (e.g., Borrell et al. 1991; Peng et al. 1999). The G allele of molecular marker *AX-89398511*, the most frequently associated SNP within this QTL, was positively associated with plant height (Touzy et al. 2019), meaning that genotypes with the G allele carry the wild-type form of *Rht-D1.* In the present study, this allele is associated to a higher number of grains for the main shoot and tillers and with earlier senescence (earlier point of inflexion and end of senescence). The effect of *Rht* genes on the number of grains per spike has often been reported. To investigate the possible causes, Miralles et al. (1988) compared isogenic lines for *Rht-D1* and *Rht-B1*, and found that the semi-dwarf alleles increased the number of fertile florets per spikelet, possibly because assimilate partitioning to the spike was more favorable than to the stem during the pre-anthesis period. Association between *Rht* genes and senescence has also already been reported (Blake et al. 2009; Camargo et al. 2016). Christopher et al. (2018) reported that earlier senescence (defined in that case as thermal time from anthesis to 10% and 50% loss of maximal greenness) was associated with the *Rht* semi-dwarfing allele in the SeriM82/Hartog population. They could not conclude whether this gene was directly associated with senescence or with linked genes. More recently, Jobson et al. (2019) compared the photosynthesis characteristics of *Rht-B1* isogenic lines in space-planted, irrigated field conditions. They concluded that the semi-dwarf allele reduced flag leaf chlorophyll content at anthesis and photosynthetic rate per unit area at anthesis and 14 days after anthesis. Despite these changes, they did not detect any significant modifications in genes global expression due to the presence of the semi-dwarfing allele that could help explain these differences. In addition, in our conditions, plants were placed at random in the greenhouse, so we cannot ignore the possibility that semi-dwarf plants were in competition for light with taller ones, which could have impacted the number of grains and senescence.

The photoperiod sensitivity gene *Ppd-D1* on chromosome 2D co-localizes with a QTL for flowering date and a QTL for the onset of senescence and the point of inflexion of the logistic curve. Co-localizations between phenology and senescence traits are widely reported (e.g., Verma et al. 2004; Bogard et al. 2011; Pinto et al. 2016; Camargo et al. 2016; Christopher et al. 2018). Verma et al. (2004) identified a QTL located on the short arm of chromosome 2D which was associated with higher yield and delayed senescence, but not with flowering date in the conditions tested. By characterizing a Beaver/Soissons bi-parental population those authors showed that the allele responsible for delayed senescence came from the Soissons cultivar. Soissons is in our panel, so we checked the polymorphism of the most strongly associated molecular marker and found a similar result. This means that earlier varieties, with the mutant version of *Ppd-D1* which is insensitive to photoperiod*,* have flag leaves that stay green for longer. Based on several studies, Bogard et al. (2011) presented different hypotheses for the relationship between wheat precocity and senescence. Three hypotheses involved the direct or indirect effect of comparing varieties differing in precocity in the same field trial. Essentially, in field experiments, terminal stresses, such as high temperature and water and nitrogen deficiencies, often occur at about the same date for all the genotypes. As senescence is highly dependent on these environmental conditions, late flowering plants would be expected to have shorter stay-green phenotypes. However, as our experiments were carried out in controlled conditions where water and nitrogen were non-limiting, those assumptions do not fit. Another hypothesis, suggested by Wingler et al. (2010) for *A. thaliana*, is that the relationship between precocity and leaf senescence is linked to a change in metabolism soon after anthesis, when the dismantling of structural components of leaves releases sugars after anthesis, which could trigger senescence.

Only one QTL on chromosome 7A was associated with TKW, but it did not co-localize with the QTL identified in our previous work (Touzy et al. 2019). However, the two studies were conducted in very different conditions. In the present study, having only one plant per pot (12 cm wide) resulted in a plant density of about 70 plants m^−2^ which is very low compared to typical autumn-sown fields (~ 200–300 plants m^−2^). The study of a complex trait such as heat tolerance is easier in controlled conditions and we assumed that sowing density and potential differences in tillering and TKW would not strongly affect the assessment of genotype behavior. For agronomical traits, such as yield components, controlled-condition studies for a breeding or agricultural context are not as representative as field experiments. As stressed by Tardieu (2012), both kinds of studies are relevant as they enable different behaviors to be accessed.

### QTL associated with the response to heat stress

Seven QTLs associated with the response to heat treatment were identified. None of them are associated with flowering time, which validated our protocol in allowing us to identify QTL linked to heat stress tolerance, without confounding effects due to escape.

Four QTLs are associated with senescence traits. On chromosome 4A, the QTL associated with the area under the curve (AUCK) is centromeric. As linkage disequilibrium is high in this region, the probability of identifying co-localization with other QTLs or genes from literature is therefore too high. For the remaining QTLs, it is relevant to check co-localization with our previous field trials of the same panel of varieties (Touzy et al. 2019). Trials from a large multi-environmental network were grouped according to their water stress scenarios: optimal, no stress, terminal stress, stress during anthesis, and stress from booting to harvest. Even though drought stress is often associated with heat stress, the heat stress component of the environments was not characterized. Three QTLs associated with the response of grain weight to heat stress were identified in the present study. Two of them co-localize with others found in the multi-environmental network study (Touzy et al. 2019). The SNP most associated with the chromosome 3B QTL was associated with grain yield in environments with water stress during the anthesis period. The chromosome 4B QTL associated with the response of main shoot TKW co-localized with a QTL associated with TKW in all the water stress scenarios and in optimal conditions. More than 14% of the variability in plant stress responses could be explained by the QTL we have detected here. We have checked reports that place QTLs for TKW and grain yield in hot conditions on chromosome 4B, many of which are reviewed by Zhang et al. (2010), Bouffier (2014), Tricker et al. (2018) and Guan et al. (2020). Pinto et al. (2010) also reported two QTLs on chromosome 4B for TKW and plant height in hot conditions. Noting that several studies had identified the latter QTL the authors suggested that this region may co-localize with the *Rht-B1* gene. However, the bi-parental Seri M82/Babax population used to identify this QTL did not segregate for any known *Rht* genes. We checked the positions of our QTL and *Rht-B1* (Online Resource 8) and found that both the physical (~ 29 Mb) and genetic (~ 40 cM) distances between them are large. Therefore, it seems likely that one or more genes linked to thermal stress tolerance are present in this area, independently of *Rht-B1*.

Even though these QTLs did not explain much of the variability in the stress response, co-localizing QTL between controlled condition and field experiments allowed us to validate our approach.

## Conclusion

We have investigated terminal heat stress tolerance using a European panel of elite winter wheat varieties. An optimal condition and a post-anthesis heat treatment applied to plants grown in the greenhouse showed how widely varieties differ in their ability to tolerate this stress. Grain weight was strongly impacted by this stress, while senescence traits were only slightly influenced. The genetic determinant of heat tolerance was determined by a GWAS. We identified a significant SNP × treatment interaction for TKW on the telomeric region of the short arm of chromosome 4B. Focusing on a well-defined terminal heat stress, we integrate our findings with other stress scenarios to help identify the genomic regions needed to develop heat stress-tolerant crops.

## Supplementary Information

Below is the link to the electronic supplementary material.

Supplementary file1 (DOCX 515 kb)

## Abbreviations

GWAS: Genome-wide association study
QTL: Quantitative trait loci
SNP: Single nucleotide polymorphism
