# Supplementary material for: Identification of QTLs affecting post-anthesis heat stress responses in European bread wheat
Source: Theor Appl Genet. 2022 Jan 5;135(3):947–64. doi: 10.1007/s00122-021-04008-5 (PMC8942932; doi:10.1007/s00122-021-04008-5)
Supplement: Supplementary file 1 — Supplementary file1 (DOCX 515 kb) [file 122_2021_4008_MOESM1_ESM.docx]

# Online Resource 1 Wheat varieties tested, year of release (YR) and country where first registered.

| Variety | YR | Origin | Variety | YR | Origin | Variety | YR | Origin |
| --- | --- | --- | --- | --- | --- | --- | --- | --- |
| ACCOR | 2007 | France | DINOSOR | 2005 | France | OREGRAIN | 2011 | France |
| ACCROC | 2010 | France | EPHOROS | 2004 | France | ORVANTIS | 2000 | France |
| ACIENDA | 2004 | France | EPIDOC | 2006 | France | OXEBO | 2010 | France |
| ACOUSTIC | 2010 | France | ESKET | 2007 | Germany | PAJERO | 1995 | Belgium |
| ADAGIO | 2009 | France | EUCLIDE | 2007 | France | PAKITO | 2010 | France |
| ADHOC | 2010 | France | EUREKA | 1992 | France | PALEDOR | 2005 | France |
| AEROBIC | 2009 | France | EXELCIOR | 2007 | France | PAPAGENO | 2007 | Austria |
| ALCAZAR | 2004 | France | EXOTIC | 2005 | France | PARADOR | 2000 | France |
| ALCHEMY | 2005 | UK | EXPERT | 2007 | France | PEPIDOR | 2007 | France |
| ALDRIC | 2007 | France | FAIRPLAY | 2011 | France | PERFECTOR | 2004 | France |
| ALIGATOR | 2010 | France | FARINELLI | 2010 | France | PHARE | 2007 | France |
| ALIXAN | 2005 | France | FIGARO | 2011 | France | PIERROT | 2009 | Danemark |
| ALIZEO | 2010 | France | FLAMENKO | 2010 | France | PIRENEO | 2005 | Austria |
| ALLEZ_Y | 2010 | France | FLAUBERT | 2010 | France | PLAYER | 2010 | France |
| ALTAMIRA | 2009 | France | FLUOR | 2010 | France | PR22R20 | 2002 | France |
| ALTIGO | 2007 | France | FOLKLOR | 2010 | France | PREMIO | 2007 | France |
| ALTRIA | 1996 | France | FORBLANC | 2009 | France | PREVERT | 2010 | France |
| AMADOR | 2010 | France | FRELON | 2000 | France | PRIMO | 2009 | Italy |
| AMBELLO | 2010 | France | GALACTIC | 2007 | France | RACINE | 2010 | France |
| AMBITION | 2004 | Danemark | GALLANT | 2007 | UK | RAZZANO | 2010 | France |
| AMUNDSEN | 2007 | France | GALOPAIN | 2009 | France | RENAN | 1989 | France |
| ANDALOU | 2001 | France | GALPINO | 2010 | Spain | RIMBAUD | 2010 | France |
| ANTONIUS | 2006 | France | GALVANO | 2008 | Danemark | ROBIGUS | 2002 | UK |
| APACHE | 1998 | France | GARANTUS | 2007 | Poland | RONSARD | 2011 | France |
| APRILIO | 2010 | France | GARCIA | 2006 | France | ROSARIO | 2004 | France |
| ARAMIS | 2009 | France | GLADIATOR | 2002 | UK | ROYSSAC | 2002 | France |
| AREZZO | 2007 | France | GLASGOW | 2004 | UK | RUBISKO | 2011 | France |
| ARISTOTE | 2010 | France | GONCOURT | 2009 | France | RUSTIC | 2005 | Belgium |
| ARKEOS | 2010 | France | GRAINDOR | 2006 | France | SAINT_EX | 2010 | France |
| ARLEQUIN | 2007 | France | HAUSSMANN | 2006 | France | SAMURAI | 2005 | France |
| ARTDECO | 2011 | France | HEREWARD | 1992 | France | SANKARA | 2004 | France |
| ASCOTT | 2011 | France | HISSEO | 2010 | Italy | SCENARIO | 2010 | France |
| ATHLON | 2010 | France | ILLICO | 2010 | France | SCOR | 2009 | France |
| ATTITUDE | 2009 | France | INNOV | 2005 | France | SEBASTO | 2007 | France |
| ATTLASS | 2005 | France | INSTINCT | 2006 | France | SELEKT | 2007 | France |
| AUBUSSON | 2001 | France | INTERET | 2007 | France | SEYRAC | 2006 | France |
| AUTAN | 2000 | France | IRIDIUM | 2007 | France | SIRTAKI | 2008 | Italy |
| AZZERTI | 2010 | France | ISENGRAIN | 1997 | France | SOBBEL | 2009 | France |
| BAGOU | 2007 | France | JAGUAR | 2009 | Italy | SOGOOD | 2006 | France |
| BAROK | 2009 | France | JB_DIEGO | 2006 | UK | SOISSONS | 1988 | France |
| BASTIDE | 2002 | France | KALAHARI | 2010 | Germany | SOKAL | 2010 | France |
| BERGAMO | 2011 | France | KALANGO | 2002 | Italy | SOLEDAD | 2008 | Italy |
| BERMUDE | 2007 | France | KALYSTAR | 2010 | France | SOLEHIO | 2009 | France |
| BIANCOR | 2010 | France | KARILLON | 2010 | France | SOLLARIO | 2007 | France |
| BOISSEAU | 2007 | France | KOMETUS | 2011 | Germany | SOLUTION | 2007 | France |
| BOLOGNA | 2002 | Spain | KORELI | 2007 | France | SOPHYTRA | 2007 | France |
| BOREGAR | 2007 | France | KWS_PODIUM | 2010 | UK | SORRIAL | 2009 | France |
| BUENNO | 2007 | France | LASER | 2010 | Italy | SPONSOR | 1995 | France |
| CAMP_REMY | 1980 | France | LAURIER | 2011 | France | SWEET | 2010 | France |
| CAPHORN | 2000 | France | LEAR | 2007 | UK | SWINGGY | 2009 | France |
| CATALAN | 2002 | France | LIMES | 2002 | France | SY_ALTEO | 2010 | France |
| CCB_INGENIO | 2006 | Spain | LORD | 2009 | France | SY_EPSON | 2011 | France |
| CELESTIN | 2010 | France | MANAGER | 2006 | France | SY_MATTIS | 2010 | France |
| CELLULE | 2011 | France | MARCELIN | 2009 | France | SY_MOISSON | 2011 | France |
| CENTENAIRE | 2002 | Belgium | MARKSMAN | 2006 | UK | SY_TOLBIAC | 2011 | France |
| CEZANNE | 1998 | France | MERCATO | 2005 | France | TAPIDOR | 2001 | France |
| CH_NARA | 2009 | Switzerland | MH_09.17 | NA | NA | TIMING | 2010 | France |
| CHARGER | 1997 | France | MIROIR | 2010 | France | TOISONDOR | 2004 | France |
| CHEVALIER | 2006 | Austria | MOSKITO | 2011 | France | TRAPEZ | 2009 | France |
| CLAIRE | 1997 | UK | MUSIK | 2010 | France | TREMIE | 1992 | France |
| COMPIL | 2010 | France | NIRVANA | 2001 | France | TULIP | 2010 | France |
| CORDIALE | 2005 | France | NUCLEO | 2010 | France | USKI | 2009 | France |
| CORVUS | 1998 | Belgium | OAKLEY | 2006 | UK | VALODOR | 2007 | France |
| COURTOT | 1974 | France | ODYSSEE | 2011 | France | VISCOUNT | 2007 | UK |
| CROISADE | 2010 | France | ORATORIO | 1996 | France | WARRIOR | 2009 | UK |
| CROUSTY | 1995 | France | ORCAS | 2010 | Germany | XI19 | 2001 | UK |
| DIALOG | 2007 | France |  |  |  |  |  |  |

# Online Resource 2 Representation of the experimental design in the greenhouse. This split-plot design has sub-plots nested into five sub-blocks nested in turn into two whole-plots arranged in three replicates on 15 tables. Whole-plot (or treatment) arrangements are shown in pale red and white. Sub-plot (or genotype) arrangements on the middle of the table are illustrated for Table 13 as an example, each table containing 120 pots. The NB1 genotype was positioned around the edge of each table to limit border effects (orange pots). Each table was split into two incomplete sub-blocks (e.g. Tables 1 and 4) in which three checks were positioned at random. Positions of thermo-buttons monitoring the temperature are shown as blue circles. The grey areas at the top and bottom left represent the air extractors.

#

# Online Resource 3 Variance components (Model 3, see Material and Methods) for each trait measured in 2016 and 2017 on 199 wheat varieties with two post-flowering temperature treatments. Mean, mean of fitted values; Sd, standard deviation for fitted values; H^2^, heritability; SI, stress index, ssT, sum of squares explained by the treatment; varG, variance explained by the genotype, varGT, variance explained by the interaction; varRes, residual variance.

| Year | Variable | | Unit | Mean | Sd | H^2^ | SI | ssT | varG | varGT | varRes |
| --- | --- | --- | --- | --- | --- | --- | --- | --- | --- | --- | --- |
| 2016 | Flowering Date | D.Z65 | d | 174.12 | 6.34 | 0.95 | 0.00 | 3.86E-04 | 26.88*** | 7.71E-07 | 7.69 |
|  | Senescence  parameters | D | µg cm^-3^ | 4.20 | 0.85 | 0.23 | 5.54 | 1.44 | 0.46^†^ | 0.7** | 7.35 |
|  |  | K | µg cm^-3^ | 45.56 | 3.05 | 0.75 | -1.31 | 21.67 | 8.94*** | 1.94E-06 | 17.85 |
|  |  | a | µg cm^-3^ | 2.32E-04 | 3.20E-04 | 1.11E-02 | -192.44 | 5.95E-06 | 6.91E-09 | 2.26E-13 | 3.71E-06 |
|  |  | r | µg cm^-3^ °Cd^-1^ | 2.66E-02 | 2.78E-03 | 0.30 | -5.31 | 3.97E-05 | 6.12e-06** | 3.76e-06* | 7.26E-05 |
|  | Senescence traits | XPI | °Cd | 613.81 | 67.29 | 0.79 | 6.89 | 19288.11* | 3174.94*** | 135.47 | 4672.52 |
|  |  | YPI | µg cm^-3^ | 26.98 | 1.85 | 0.75 | -0.21 | 8.23E-03 | 2.87*** | 0.35** | 4.58 |
|  |  | VIT | µg cm^-3.^ °Cd^-1^ | -0.30 | 0.04 | 0.50 | -7.38 | 6.69E-03 | 1.51e-03*** | 4.88e-04* | 0.01 |
|  |  | START | °Cd | 516.95 | 68.25 | 0.72 | 7.54 | 10990.11 | 3162.87*** | 583.09** | 5594.55 |
|  |  | fSTART | µg cm^-3^ | 45.58 | 3.06 | 0.84 | -0.62 | 1.19 | 8.13*** | 0.17 | 8.88 |
|  |  | END | °Cd | 710.67 | 67.87 | 0.77 | 6.44 | 54812.36** | 3297.82*** | 2.69E-04 | 5921.87 |
|  |  | fEND | µg cm^-3^ | 8.38 | 0.91 | 0.29 | 2.14 | 0.77 | 0.55* | 0.65** | 6.16 |
|  |  | AUC | µg cm^-3^ °Cd | 24901.76 | 3694.25 | 0.82 | 5.82 | 22360141.43 | 10314251.87*** | 6.98 | 13856378.54 |
|  |  | AUCK | µg cm^-3^ °Cd | 30852.30 | 4677.40 | 0.85 | 6.41 | 29393172.45 | 15566752.51*** | 9.04 | 15901778.66 |
|  | Number of spikes | SPP | plant^-1^ | 4.54 | 1.08 | 0.78 | 3.14 | 0.83 | 0.94*** | 0.05 | 1.45 |
|  | Main shoot | NbGrain |  | 42.18 | 7.62 | 0.82 | 5.13 | 150.86 | 47.03*** | 1.47 | 58.79 |
|  |  | GW | g | 1.71 | 0.44 | 0.73 | 29.31 | 2.91*** | 0.07*** | 0.01*** | 0.11 |
|  |  | TKW | g | 40.92 | 7.52 | 0.66 | 25.73 | 1721.39*** | 15.02*** | 4.35*** | 33.97 |
|  |  | Grain.Area | mm² | 14.89 | 1.64 | 0.74 | 15.28 | 75.15*** | 0.97*** | 0.15*** | 1.54 |
|  |  | Grain.Width | mm | 3.46 | 0.25 | 0.69 | 11.05 | 1.92*** | 0.02*** | 4.21e-03*** | 0.03 |
|  |  | Grain.Length | mm | 5.90 | 0.27 | 0.85 | 4.30 | 1.16*** | 0.05*** | 2.84e-03** | 0.04 |
|  | Tillers | NbGrain |  | 139.71 | 37.62 | 0.78 | 8.26 | 6179.54. | 1176.72*** | 51.9 | 1795.69 |
|  |  | GW | g | 5.52 | 1.68 | 0.71 | 26.51 | 47.17*** | 1.54*** | 0.21** | 3.09 |
|  |  | TKW | g | 39.79 | 6.50 | 0.69 | 20.40 | 730.52*** | 13.13*** | 2.98*** | 26.30 |
|  |  | Grain.Area | mm² | 14.25 | 1.50 | 0.77 | 12.88 | 35.23*** | 0.93*** | 0.14*** | 1.28 |
|  |  | Grain.Width | mm | 3.40 | 0.22 | 0.73 | 8.44 | 0.91*** | 0.02*** | 2.81e-03*** | 0.03 |
|  |  | Grain.Length | mm | 5.77 | 0.28 | 0.84 | 4.65 | 0.74*** | 0.05*** | 5.28e-03*** | 0.04 |
| 2017 | Flowering date | D.Z65 | d | 177.22 | 6.96 | 0.95 | -0.12 | 3.41 | 32.49*** | 1.08E-06 | 10.49 |
|  | Senescence  parameter | D | µg cm^-3^ | 6.00 | 0.43 | 0.13 | -1.01 | 0.38 | 0.13^†^ | 2.15E-07 | 4.97 |
|  |  | K | µg cm^-3^ | 46.72 | 3.11 | 0.79 | 0.78 | 19.02 | 10.55*** | 4.26E-06 | 16.58 |
|  |  | a | µg cm^-3^ | 1.00E-03 | 4.78E-04 | 4.24E-02 | -8.66 | 9.09E-07 | 2.66E-07 | 2.74E-07 | 3.55E-05 |
|  |  | r | µg cm^-3^ °Cd^-1^ | 1.89E-02 | 3.24E-03 | 0.45 | 3.50 | 9.27E-06 | 8.77e-06*** | 7.67E-07 | 6.20E-05 |
|  | Stay-green trait | XPI | °Cd | 700.63 | 71.69 | 0.77 | 0.98 | 1388.29 | 5385.5*** | 401.45 | 8465.80 |
|  |  | YPI | µg cm^-3^ | 29.36 | 1.52 | 0.80 | 0.35 | 1.34 | 2.58*** | 4.03E-07 | 3.97 |
|  |  | VIT | µg cm^-3.^ °Cd^-1^ | -0.22 | 0.04 | 0.54 | 4.50 | 1.71E-03 | 1.65e-03*** | 1.24E-04 | 0.01 |
|  |  | START | °Cd | 547.95 | 61.62 | 0.75 | 3.38 | 14542.59 | 3890.84*** | 2.31E-03 | 7943.47 |
|  |  | fSTART | µg cm^-3^ | 48.43 | 2.83 | 0.85 | 0.50 | 9.96 | 8.54*** | 9.33E-07 | 9.20 |
|  |  | END | °Cd | 853.31 | 90.27 | 0.70 | -0.59 | 630.34 | 8468.4*** | 788 | 19708.98 |
|  |  | fEND | µg cm^-3^ | 10.29 | 0.41 | 0.20 | -0.23 | 0.06 | 0.17* | 2.41E-06 | 4.17 |
|  |  | AUC | µg cm^-3^ °Cd | 28782.44 | 3630.59 | 0.81 | 2.00 | 17372593.86 | 13775145.15*** | 86987 | 19515502.45 |
|  |  | AUCK | µg cm^-3^ °Cd | 37491.58 | 4634.85 | 0.82 | 1.54 | 8239642.45 | 21997032.13*** | 1136065.62 | 25439236.26 |
|  | Number of sspikes | SPP | plant^-1^ | 5.04 | 0.93 | 0.71 | 4.85 | 7.89* | 0.81*** | 0.02 | 1.90 |
|  | Main shoot | NbGrain |  | 36.74 | 7.50 | 0.77 | 9.25 | 144.31 | 43.12*** | 8.03*** | 54.69 |
|  |  | GW | g | 1.38 | 0.38 | 0.72 | 27.94 | 1.8*** | 0.07*** | 0.02*** | 0.10 |
|  |  | TKW | g | 37.40 | 6.57 | 0.71 | 21.88 | 773.45*** | 18.16*** | 5.35*** | 29.48 |
|  |  | Grain.Area | mm² | 14.20 | 1.57 | 0.74 | 13.48 | 34.57*** | 1.14*** | 0.33*** | 1.46 |
|  |  | Grain.Width | mm | 3.39 | 0.23 | 0.70 | 8.93 | 0.88*** | 0.02*** | 7.25e-03*** | 0.03 |
|  |  | Grain.Length | mm | 5.78 | 0.29 | 0.83 | 4.78 | 0.8*** | 0.05*** | 7.43e-03*** | 0.04 |
|  | Tillers | NbGrain |  | 136.33 | 31.36 | 0.70 | 13.92 | 17145.46** | 808.25*** | 71.88 | 1883.59 |
|  |  | GW | g | 5.21 | 1.52 | 0.73 | 25.87 | 80.68*** | 1.47*** | 0.14 | 2.92 |
|  |  | TKW | g | 38.01 | 5.36 | 0.78 | 14.49 | 255.4*** | 15.39*** | 4.05*** | 14.08 |
|  |  | Grain.Area | mm² | 13.66 | 1.28 | 0.81 | 8.35 | 12.08*** | 1.06*** | 0.21*** | 0.87 |
|  |  | Grain.Width | mm | 3.35 | 0.16 | 0.76 | 4.81 | 0.24*** | 0.02*** | 4.37e-03*** | 0.02 |
|  |  | Grain.Length | mm | 5.65 | 0.28 | 0.88 | 3.66 | 0.48*** | 0.06*** | 5.33e-03*** | 0.03 |

*Wald and LTR test: ^***^P-values < 0.001; ^**^P-values < 0.01; ^*^P-values < 0.05 and ^†^P-values < 0.1*

#

# Online Resource 4 Genotypes TKW (g) in no stress (NS) and stress (S) post-flowering temperature treatments (with standard deviation), the coefficient of Genotype × Treatment interaction using Model 9 (the lower the value the lower the tolerance to the treatment), and the Stress Index for each genotype (SIg) calculated as Equation 8.

| GENOTYPE | NS | |  | S | | Genotype by Treatment | SIg |
| --- | --- | --- | --- | --- | --- | --- | --- |
|  | gBLUP | gBLUP_sd |  | gBLUP | gBLUP_sd |  |  |
| ACCOR | 33.28 | 1.95 |  | 27.4 | 1.95 | 1.25 | 17.67% |
| ACCROC | 40.23 | 1.98 |  | 33.12 | 2.09 | 1.44 | 17.67% |
| ACIENDA | 35.91 | 1.91 |  | 28.93 | 1.91 | 0.97 | 19.44% |
| ACOUSTIC | 40.62 | 1.89 |  | 29.2 | 1.89 | -0.94 | 28.11% |
| ADAGIO | 44.76 | 1.87 |  | 32.01 | 1.86 | -1.16 | 28.49% |
| ADHOC | 42.95 | 1.97 |  | 34.03 | 1.97 | 0.77 | 20.77% |
| AEROBIC | 44.91 | 1.94 |  | 36.92 | 1.94 | 1.54 | 17.79% |
| ALCAZAR | 46.71 | 1.92 |  | 32.81 | 2 | -1.56 | 29.76% |
| ALCHEMY | 46.29 | 2.02 |  | 36.76 | 1.92 | 0.85 | 20.59% |
| ALDRIC | 40.65 | 1.9 |  | 31.67 | 1.9 | 0.44 | 22.09% |
| ALIGATOR | 40.18 | 1.97 |  | 29.8 | 1.97 | -0.41 | 25.83% |
| ALIXAN | 37.05 | 1.95 |  | 28.04 | 1.95 | -0.03 | 24.32% |
| ALIZEO | 45.69 | 1.92 |  | 31.99 | 1.92 | -1.58 | 29.98% |
| ALLEZ_Y | 46.14 | 1.95 |  | 34.29 | 1.95 | -0.48 | 25.68% |
| ALTAMIRA | 45.32 | 1.91 |  | 38.16 | 1.92 | 2.06 | 15.80% |
| ALTIGO | 48.92 | 1.97 |  | 39.71 | 1.97 | 1.37 | 18.83% |
| ALTRIA | 44.99 | 1.96 |  | 34.59 | 1.96 | 0.19 | 23.12% |
| AMADOR | 34.94 | 1.92 |  | 28.94 | 1.92 | 1.39 | 17.17% |
| AMBELLO | 50.16 | 1.86 |  | 37.06 | 1.86 | -0.67 | 26.12% |
| AMBITION | 44.82 | 2.08 |  | 33.38 | 1.97 | -0.42 | 25.52% |
| AMUNDSEN | 42.3 | 1.87 |  | 28.4 | 1.87 | -2.12 | 32.86% |
| ANDALOU | 45.08 | 1.93 |  | 34.24 | 1.93 | -0.04 | 24.05% |
| ANTONIUS | 41.78 | 1.87 |  | 32.17 | 1.87 | 0.23 | 23.00% |
| APACHE | 47.64 | 1.78 |  | 38.74 | 1.72 | 1.37 | 18.68% |
| APRILIO | 44.31 | 1.97 |  | 35.27 | 2.08 | 0.87 | 20.40% |
| ARAMIS | 45.94 | 1.91 |  | 35.97 | 1.91 | 0.56 | 21.70% |
| AREZZO | 47.16 | 1.9 |  | 36.88 | 1.9 | 0.54 | 21.80% |
| ARISTOTE | 41.45 | 1.94 |  | 32.57 | 1.94 | 0.6 | 21.42% |
| ARKEOS | 31.88 | 1.94 |  | 26.39 | 1.94 | 1.29 | 17.22% |
| ARLEQUIN | 38.05 | 1.89 |  | 26.69 | 1.89 | -1.24 | 29.86% |
| ARTDECO | 40.77 | 1.99 |  | 37.07 | 1.99 | 3.43 | 9.08% |
| ASCOTT | 42.77 | 1.9 |  | 29.98 | 1.9 | -1.44 | 29.90% |
| ATHLON | 46.34 | 1.93 |  | 33.69 | 1.92 | -0.91 | 27.30% |
| ATTITUDE | 42.33 | 1.96 |  | 29.5 | 2.06 | -1.52 | 30.31% |
| ATTLASS | 46.69 | 1.93 |  | 30.7 | 1.93 | -2.74 | 34.25% |
| AUBUSSON | 42.35 | 1.89 |  | 32.62 | 1.89 | 0.23 | 22.98% |
| AUTAN | 52.82 | 1.97 |  | 41.59 | 1.97 | 0.72 | 21.26% |
| AZZERTI | 41.58 | 1.98 |  | 32.51 | 1.98 | 0.51 | 21.81% |
| BAGOU | 39.17 | 2.03 |  | 28.86 | 1.93 | -0.5 | 26.32% |
| BAROK | 42.63 | 1.97 |  | 30.66 | 2.07 | -1 | 28.08% |
| BASTIDE | 42.6 | 2.43 |  | 31.9 | 2.43 | -0.28 | 25.12% |
| BERGAMO | 44.41 | 1.93 |  | 36.13 | 1.93 | 1.31 | 18.64% |
| BERMUDE | 41.43 | 1.07 |  | 32.38 | 1.08 | 0.5 | 21.84% |
| BIANCOR | 42.36 | 1.94 |  | 32.73 | 2.03 | 0.29 | 22.73% |
| BOISSEAU | 46.55 | 1.96 |  | 35.67 | 2.06 | 0.12 | 23.37% |
| BOLOGNA | 37.74 | 1.97 |  | 30.54 | 1.97 | 1.07 | 19.08% |
| BOREGAR | 45.8 | 1.08 |  | 35.91 | 1.08 | 0.59 | 21.59% |
| BUENNO | 46.76 | 1.97 |  | 41.06 | 1.97 | 3.07 | 12.19% |
| CAMP_REMY | 44.08 | 1.98 |  | 33.65 | 1.98 | 0.06 | 23.66% |
| CAPHORN | 43.16 | 1.74 |  | 34.07 | 1.75 | 0.7 | 21.06% |
| CATALAN | 44.86 | 1.97 |  | 33.94 | 1.98 | -0.11 | 24.34% |
| CCB_INGENIO | 50.09 | 2 |  | 39.1 | 2.11 | 0.51 | 21.94% |
| CELESTIN | 47.95 | 1.94 |  | 37.97 | 2.04 | 0.81 | 20.81% |
| CELLULE | 46.35 | 1.97 |  | 37.09 | 1.97 | 1.01 | 19.98% |
| CENTENAIRE | 47.04 | 1.9 |  | 35.91 | 1.9 | 0.05 | 23.66% |
| CEZANNE | 57.15 | 1.97 |  | 46.04 | 1.97 | 1.34 | 19.44% |
| CH_NARA | 42.99 | 2.02 |  | 32.09 | 2.02 | -0.34 | 25.35% |
| CHARGER | 43.67 | 1.74 |  | 31.38 | 1.75 | -1.04 | 28.14% |
| CHEVALIER | 47.29 | 1.93 |  | 32.35 | 2.04 | -2.07 | 31.59% |
| CLAIRE | 42.17 | 1.83 |  | 29.88 | 1.83 | -1.23 | 29.14% |
| COMPIL | 40.5 | 1.88 |  | 31.27 | 1.87 | 0.28 | 22.79% |
| CORDIALE | 43.35 | 1.92 |  | 30.89 | 1.92 | -1.18 | 28.74% |
| CORVUS | 40.65 | 1.91 |  | 27.09 | 2 | -2.15 | 33.36% |
| COURTOT | 41.82 | 2.14 |  | 36.78 | 2.02 | 2.81 | 12.05% |
| CROISADE | 44.03 | 1.93 |  | 36.37 | 1.93 | 1.62 | 17.40% |
| CROUSTY | 46.61 | 1.97 |  | 36.64 | 1.97 | 0.64 | 21.39% |
| DIALOG | 45.8 | 1.97 |  | 31.98 | 2.08 | -1.63 | 30.17% |
| DINOSOR | 46.25 | 1.91 |  | 34.78 | 1.91 | -0.25 | 24.80% |
| EPHOROS | 45.37 | 1.92 |  | 34.41 | 1.92 | -0.08 | 24.16% |
| EPIDOC | 43.87 | 2.08 |  | 30.98 | 1.97 | -1.35 | 29.38% |
| ESKET | 40.41 | 1.88 |  | 30.88 | 1.97 | 0.1 | 23.58% |
| EUCLIDE | 45.29 | 1.92 |  | 36.62 | 2.01 | 1.21 | 19.14% |
| EUREKA | 52 | 1.99 |  | 43.03 | 2 | 1.89 | 17.25% |
| EXELCIOR | 45.87 | 2.48 |  | 38.32 | 2.6 | 1.91 | 16.46% |
| EXOTIC | 46.71 | 1.92 |  | 38.31 | 1.92 | 1.54 | 17.98% |
| EXPERT | 56.57 | 1.91 |  | 40.43 | 1.91 | -1.57 | 28.53% |
| FAIRPLAY | 42.56 | 1.93 |  | 30.29 | 1.93 | -1.17 | 28.83% |
| FARINELLI | 49.15 | 1.91 |  | 40.14 | 1.91 | 1.51 | 18.33% |
| FIGARO | 50.68 | 1.98 |  | 44.44 | 1.98 | 3.27 | 12.31% |
| FLAMENKO | 42.2 | 1.96 |  | 33.77 | 1.96 | 0.95 | 19.98% |
| FLAUBERT | 38.33 | 1.94 |  | 27.35 | 1.94 | -0.98 | 28.65% |
| FLUOR | 39.22 | 1.94 |  | 30.29 | 1.94 | 0.29 | 22.77% |
| FOLKLOR | 46.02 | 1.99 |  | 33.85 | 2.1 | -0.67 | 26.45% |
| FORBLANC | 49.01 | 1.97 |  | 40.41 | 1.89 | 1.72 | 17.55% |
| FRELON | 42.23 | 1.82 |  | 33.03 | 1.82 | 0.52 | 21.79% |
| GALACTIC | 44.97 | 2.12 |  | 32.87 | 1.93 | -0.77 | 26.91% |
| GALLANT | 38.15 | 1.91 |  | 28.21 | 2.01 | -0.42 | 26.06% |
| GALOPAIN | 40.74 | 1.87 |  | 32.11 | 1.87 | 0.65 | 21.18% |
| GALPINO | 42.59 | 1.87 |  | 34.26 | 1.87 | 1.05 | 19.56% |
| GALVANO | 41.77 | 1.91 |  | 29.92 | 1.91 | -1.04 | 28.37% |
| GARANTUS | 38.34 | 1.87 |  | 28.11 | 1.88 | -0.56 | 26.68% |
| GARCIA | 45.04 | 1.93 |  | 34.04 | 1.93 | -0.14 | 24.42% |
| GLADIATOR | 42.96 | 1.92 |  | 29.43 | 1.92 | -1.83 | 31.49% |
| GLASGOW | 37.96 | 1.97 |  | 27.97 | 1.97 | -0.47 | 26.32% |
| GONCOURT | 42.27 | 2 |  | 32.42 | 2 | 0.16 | 23.30% |
| GRAINDOR | 49.38 | 1.88 |  | 41.29 | 1.88 | 2.06 | 16.38% |
| HAUSSMANN | 42.33 | 1.81 |  | 32.06 | 1.81 | -0.07 | 24.26% |
| HEREWARD | 44.13 | 1.91 |  | 30.32 | 1.91 | -1.84 | 31.29% |
| HISSEO | 44.92 | 1.91 |  | 36.88 | 2 | 1.52 | 17.90% |
| ILLICO | 48.13 | 1.86 |  | 41.49 | 1.86 | 2.71 | 13.80% |
| INNOV | 49.06 | 1.91 |  | 39.8 | 2 | 1.35 | 18.87% |
| INSTINCT | 39.39 | 1.96 |  | 25.35 | 2.2 | -2.57 | 35.64% |
| INTERET | 47.19 | 2.05 |  | 36.52 | 1.95 | 0.32 | 22.61% |
| IRIDIUM | 48.4 | 1.92 |  | 36.02 | 1.92 | -0.49 | 25.58% |
| ISENGRAIN | 45.12 | 1.78 |  | 36.89 | 1.77 | 1.43 | 18.24% |
| JAGUAR | 36.32 | 1.96 |  | 30.22 | 2.06 | 1.51 | 16.80% |
| JB_DIEGO | 43.19 | 1.9 |  | 29.8 | 1.9 | -1.72 | 31.00% |
| KALAHARI | 46.39 | 1.95 |  | 36.72 | 1.95 | 0.78 | 20.85% |
| KALANGO | 41.61 | 1.99 |  | 32.99 | 1.99 | 0.76 | 20.72% |
| KALYSTAR | 43.78 | 1.91 |  | 31.09 | 1.91 | -1.25 | 28.99% |
| KARILLON | 44.47 | 1.95 |  | 32.52 | 1.95 | -0.75 | 26.87% |
| KOMETUS | 45.05 | 2.08 |  | 28.69 | 1.97 | -3.16 | 36.32% |
| KORELI | 48.66 | 2.01 |  | 36.94 | 2.13 | -0.09 | 24.09% |
| KWS_PODIUM | 45.32 | 1.96 |  | 34.92 | 1.96 | 0.23 | 22.95% |
| LASER | 40.39 | 1.98 |  | 30.38 | 1.98 | -0.17 | 24.78% |
| LAURIER | 55.06 | 1.97 |  | 40.62 | 1.96 | -0.8 | 26.23% |
| LEAR | 43.19 | 1.92 |  | 33.27 | 1.92 | 0.23 | 22.97% |
| LIMES | 53.15 | 1.82 |  | 39.19 | 1.82 | -0.77 | 26.27% |
| LORD | 43.96 | 1.96 |  | 37.69 | 1.96 | 2.39 | 14.26% |
| MANAGER | 44.08 | 1.95 |  | 32.16 | 1.95 | -0.78 | 27.04% |
| MARCELIN | 47.76 | 1.88 |  | 36.28 | 1.89 | -0.06 | 24.04% |
| MARKSMAN | 40.34 | 1.82 |  | 28.25 | 1.82 | -1.35 | 29.97% |
| MERCATO | 48.16 | 1.84 |  | 36.71 | 1.84 | 0 | 23.77% |
| MH_09.17 | 41.65 | 1.93 |  | 30.66 | 1.93 | -0.57 | 26.39% |
| MIROIR | 46.4 | 1.93 |  | 38.26 | 2.02 | 1.65 | 17.54% |
| MOSKITO | 46.58 | 1.9 |  | 34.93 | 1.9 | -0.31 | 25.01% |
| MUSIK | 41.36 | 1.99 |  | 33.74 | 1.99 | 1.3 | 18.42% |
| NIRVANA | 45.97 | 1.83 |  | 34.36 | 1.83 | -0.36 | 25.26% |
| NUCLEO | 43.62 | 1.9 |  | 32.36 | 1.9 | -0.47 | 25.81% |
| OAKLEY | 41.42 | 2.02 |  | 26.36 | 1.92 | -2.89 | 36.36% |
| ODYSSEE | 44.72 | 1.99 |  | 34.81 | 1.98 | 0.43 | 22.16% |
| ORATORIO | 39.47 | 2 |  | 28.11 | 1.91 | -1.05 | 28.78% |
| ORCAS | 46.69 | 1.92 |  | 36.04 | 1.93 | 0.27 | 22.81% |
| OREGRAIN | 44.59 | 1.85 |  | 35.33 | 1.93 | 0.78 | 20.77% |
| ORVANTIS | 50.27 | 1.85 |  | 37.5 | 1.85 | -0.47 | 25.40% |
| OXEBO | 48.51 | 1.97 |  | 35.74 | 1.97 | -0.7 | 26.32% |
| PAJERO | 41.23 | 1.97 |  | 32.8 | 1.97 | 0.83 | 20.45% |
| PAKITO | 49.02 | 1.89 |  | 38.91 | 1.89 | 0.87 | 20.62% |
| PALEDOR | 38.57 | 2.06 |  | 29.46 | 1.96 | 0.1 | 23.62% |
| PAPAGENO | 41.54 | 1.99 |  | 30.26 | 1.99 | -0.74 | 27.15% |
| PARADOR | 46.08 | 2.04 |  | 33.03 | 1.94 | -1.17 | 28.32% |
| PEPIDOR | 43.39 | 1.91 |  | 30.95 | 1.91 | -1.16 | 28.67% |
| PERFECTOR | 46.57 | 2.55 |  | 35.32 | 2.43 | -0.09 | 24.16% |
| PHARE | 38.69 | 1.84 |  | 26.82 | 1.92 | -1.44 | 30.68% |
| PIERROT | 42.48 | 1.92 |  | 31.04 | 2.02 | -0.71 | 26.93% |
| PIRENEO | 42.53 | 1.87 |  | 32.75 | 1.87 | 0.23 | 23.00% |
| PLAYER | 43.41 | 2.08 |  | 29.22 | 2.08 | -2.15 | 32.69% |
| PR22R20 | 41.77 | 2.07 |  | 29.64 | 1.97 | -1.19 | 29.04% |
| PREMIO | 49.37 | 1.09 |  | 37.65 | 1.07 | 0 | 23.74% |
| PREVERT | 45.72 | 1.85 |  | 36.02 | 1.84 | 0.68 | 21.22% |
| PRIMO | 34.11 | 1.92 |  | 28.16 | 2.02 | 1.32 | 17.44% |
| RACINE | 40.18 | 1.9 |  | 27.29 | 1.9 | -1.82 | 32.08% |
| RAZZANO | 51.16 | 1.91 |  | 36.69 | 1.9 | -1.31 | 28.28% |
| RENAN | 48.62 | 2.01 |  | 36.14 | 2.01 | -0.52 | 25.67% |
| RIMBAUD | 43.95 | 1.9 |  | 36.32 | 1.9 | 1.62 | 17.36% |
| ROBIGUS | 39.14 | 1.83 |  | 27.06 | 1.83 | -1.5 | 30.86% |
| RONSARD | 45.62 | 1.99 |  | 30.75 | 1.99 | -2.25 | 32.60% |
| ROSARIO | 44.24 | 1.8 |  | 33.28 | 1.8 | -0.22 | 24.77% |
| ROYSSAC | 45.55 | 1.89 |  | 32.99 | 1.89 | -0.95 | 27.57% |
| RUBISKO | 52.48 | 1.87 |  | 39.66 | 1.88 | -0.22 | 24.43% |
| RUSTIC | 46.24 | 1.94 |  | 33.28 | 1.94 | -1.09 | 28.03% |
| SAINT_EX | 39.07 | 1.99 |  | 34.45 | 1.9 | 2.7 | 11.82% |
| SAMURAI | 50.17 | 1.91 |  | 38.21 | 1.91 | -0.02 | 23.84% |
| SANKARA | 40.28 | 2.01 |  | 30.13 | 1.91 | -0.27 | 25.20% |
| SCENARIO | 40.76 | 1.97 |  | 28.81 | 1.88 | -1.22 | 29.32% |
| SCOR | 46.99 | 1.89 |  | 35.46 | 1.88 | -0.19 | 24.54% |
| SEBASTO | 46.07 | 1.89 |  | 32.22 | 1.89 | -1.61 | 30.06% |
| SELEKT | 41.47 | 1.82 |  | 29.06 | 1.82 | -1.39 | 29.93% |
| SEYRAC | 43.84 | 1.93 |  | 34.82 | 1.93 | 0.82 | 20.57% |
| SIRTAKI | 44.66 | 1.91 |  | 32.33 | 1.91 | -0.94 | 27.61% |
| SOBBEL | 55.67 | 1.93 |  | 46.77 | 1.93 | 2.4 | 15.99% |
| SOGOOD | 49.13 | 1.81 |  | 34.71 | 1.81 | -1.54 | 29.35% |
| SOISSONS | 42.81 | 1.86 |  | 35.48 | 1.86 | 1.64 | 17.12% |
| SOKAL | 43.3 | 1.95 |  | 28.86 | 1.95 | -2.3 | 33.35% |
| SOLEDAD | 45.01 | 1.87 |  | 36.3 | 1.87 | 1.15 | 19.35% |
| SOLEHIO | 50.04 | 1.95 |  | 42.54 | 1.95 | 2.47 | 14.99% |
| SOLLARIO | 42.87 | 1.96 |  | 32.86 | 1.96 | 0.14 | 23.35% |
| SOLUTION | 38.35 | 1.87 |  | 26.58 | 1.87 | -1.42 | 30.69% |
| SOPHYTRA | 52.66 | 1.82 |  | 39.49 | 1.82 | -0.39 | 25.01% |
| SORRIAL | 46.45 | 1.88 |  | 37.48 | 1.81 | 1.19 | 19.31% |
| SPONSOR | 35.51 | 2.04 |  | 24.27 | 1.94 | -1.49 | 31.65% |
| SWEET | 54.57 | 1.9 |  | 41.11 | 1.9 | -0.31 | 24.67% |
| SWINGGY | 47.75 | 1.92 |  | 32.54 | 1.92 | -2.17 | 31.85% |
| SY_ALTEO | 55.25 | 1.87 |  | 43.47 | 1.87 | 0.73 | 21.32% |
| SY_EPSON | 42.69 | 1.91 |  | 32.1 | 1.91 | -0.21 | 24.81% |
| SY_MATTIS | 43.73 | 1.92 |  | 34.75 | 1.92 | 0.84 | 20.54% |
| SY_MOISSON | 43.25 | 1.93 |  | 33.78 | 2.02 | 0.5 | 21.90% |
| SY_TOLBIAC | 47.2 | 1.9 |  | 36.97 | 1.9 | 0.57 | 21.67% |
| TAPIDOR | 37.55 | 1.89 |  | 27.51 | 1.9 | -0.55 | 26.74% |
| TIMING | 44.79 | 2 |  | 37.11 | 2.11 | 1.7 | 17.15% |
| TOISONDOR | 41.41 | 1.97 |  | 31.91 | 1.97 | 0.25 | 22.94% |
| TRAPEZ | 53.16 | 1.94 |  | 37.83 | 1.94 | -1.55 | 28.84% |
| TREMIE | 40.39 | 1.78 |  | 30.98 | 1.78 | 0.16 | 23.30% |
| TULIP | 44.26 | 1.97 |  | 31.14 | 1.97 | -1.43 | 29.64% |
| USKI | 47.98 | 1.95 |  | 37.58 | 1.95 | 0.57 | 21.68% |
| VALODOR | 48.14 | 1.81 |  | 37.9 | 1.81 | 0.68 | 21.27% |
| VISCOUNT | 43.18 | 1.92 |  | 33.94 | 1.92 | 0.61 | 21.40% |
| WARRIOR | 36.43 | 1.94 |  | 25.39 | 2.04 | -1.26 | 30.30% |
| XI19 | 37.24 | 1.99 |  | 29.36 | 2.11 | 0.62 | 21.16% |


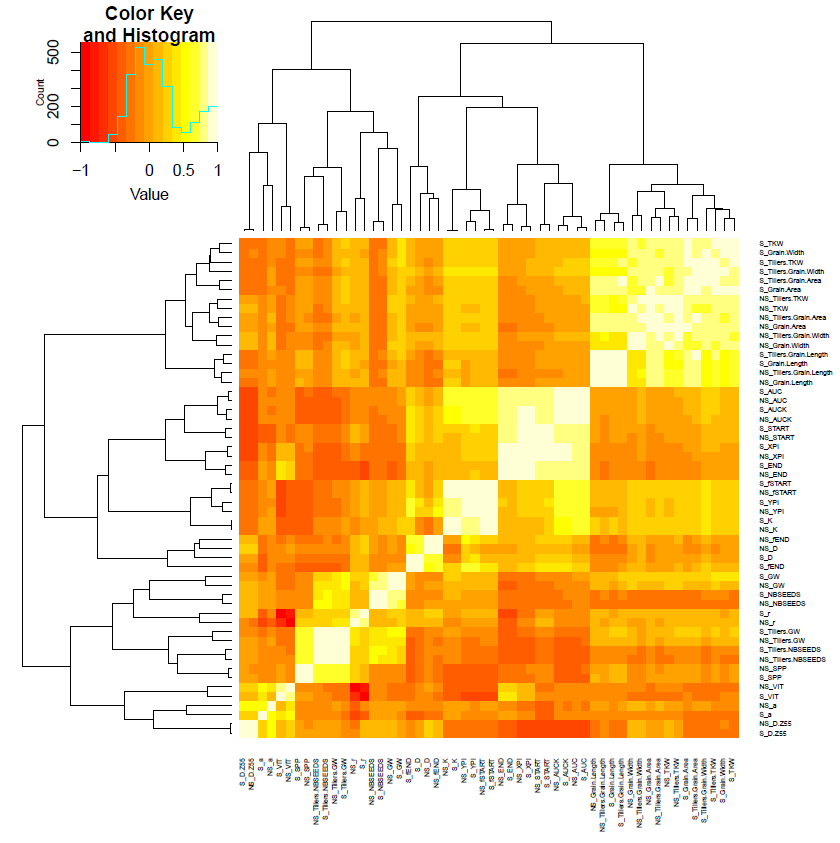


# Online Resource 5 Correlation matrix for gBLUP of all the traits scored on 199 wheat varieties in two post-flowering temperature treatments. Colors are a function of Pearson’s pairwise correlations. Treatments and traits are separated by an underscore (NS, no stress; S, stress; see Material and Methods for other abbreviations). Rows and columns are ranked as a function of their Euclidian distance.

# Online Resource 6 Correlation matrix for the Stress Index (SIg, Equation 8) of all the traits scored on 199 wheat varieties in two post-flowering temperature treatments (see Material and Methods for abbreviations).

|  | AUC | AUCK | D.Z55 | END | fEND | fSTART | Grain.Area | Grain.Length | Grain.Width | SPP | START | TKW | VIT | XPI | YPI |
| --- | --- | --- | --- | --- | --- | --- | --- | --- | --- | --- | --- | --- | --- | --- | --- |
| AUC | 1 | 0.89 | -0.21 | 0.75 | -0.14 | 0.42 | 0.26 | 0.22 | 0.29 | -0.08 | 0.63 | 0.34 | -0.18 | 0.86 | 0.16 |
| AUCK | 0.89 | 1 | -0.23 | 0.85 | 0.28 | 0.55 | 0.30 | 0.26 | 0.31 | -0.19 | 0.61 | 0.34 | -0.34 | 0.92 | 0.48 |
| D.Z55 | -0.21 | -0.23 | 1 | -0.14 | -0.01 | -0.11 | 0.00 | 0.08 | -0.02 | 0.02 | -0.13 | -0.10 | -0.02 | -0.17 | -0.10 |
| END | 0.75 | 0.85 | -0.14 | 1 | 0.13 | 0.36 | 0.24 | 0.19 | 0.27 | -0.18 | 0.32 | 0.29 | -0.68 | 0.88 | 0.24 |
| fEND | -0.14 | 0.28 | -0.01 | 0.13 | 1 | 0.44 | 0.23 | 0.21 | 0.23 | -0.22 | 0.04 | 0.19 | -0.20 | 0.10 | 0.83 |
| fSTART | 0.42 | 0.55 | -0.11 | 0.36 | 0.44 | 1 | 0.52 | 0.49 | 0.52 | -0.02 | -0.06 | 0.49 | -0.24 | 0.23 | 0.85 |
| Grain.Area | 0.26 | 0.30 | 0.00 | 0.24 | 0.23 | 0.52 | 1 | 0.89 | 0.97 | -0.10 | 0.00 | 0.95 | -0.25 | 0.16 | 0.43 |
| Grain.Length | 0.22 | 0.26 | 0.08 | 0.19 | 0.21 | 0.49 | 0.89 | 1 | 0.76 | -0.09 | -0.02 | 0.77 | -0.21 | 0.12 | 0.38 |
| Grain.Width | 0.29 | 0.31 | -0.02 | 0.27 | 0.23 | 0.52 | 0.97 | 0.76 | 1 | -0.11 | 0.00 | 0.95 | -0.28 | 0.19 | 0.42 |
| SPP | -0.08 | -0.19 | 0.02 | -0.18 | -0.22 | -0.02 | -0.10 | -0.09 | -0.11 | 1 | -0.16 | -0.10 | 0.09 | -0.20 | -0.14 |
| START | 0.63 | 0.61 | -0.13 | 0.32 | 0.04 | -0.06 | 0.00 | -0.02 | 0.00 | -0.16 | 1 | 0.06 | 0.26 | 0.72 | -0.02 |
| TKW | 0.34 | 0.34 | -0.10 | 0.29 | 0.19 | 0.49 | 0.95 | 0.77 | 0.95 | -0.10 | 0.06 | 1 | -0.25 | 0.23 | 0.39 |
| VIT | -0.18 | -0.34 | -0.02 | -0.68 | -0.20 | -0.24 | -0.25 | -0.21 | -0.28 | 0.09 | 0.26 | -0.25 | 1 | -0.35 | -0.21 |
| XPI | 0.86 | 0.92 | -0.17 | 0.88 | 0.10 | 0.23 | 0.16 | 0.12 | 0.19 | -0.20 | 0.72 | 0.23 | -0.35 | 1 | 0.16 |
| YPI | 0.16 | 0.48 | -0.10 | 0.24 | 0.83 | 0.85 | 0.43 | 0.38 | 0.42 | -0.14 | -0.02 | 0.39 | -0.21 | 0.16 | 1 |


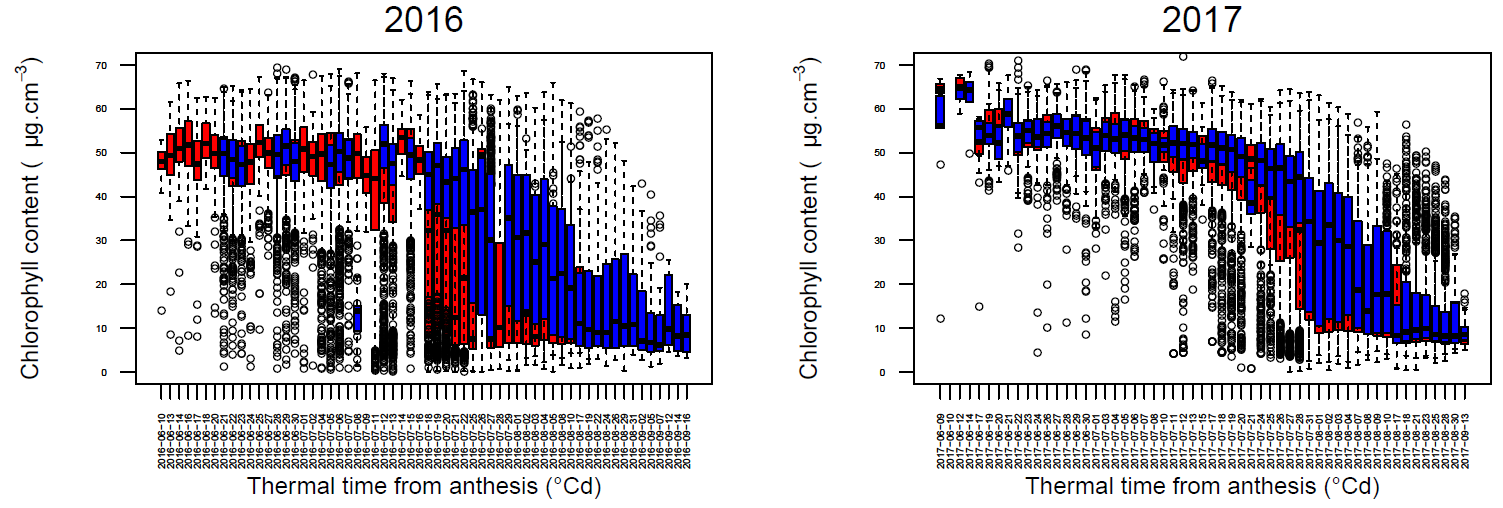


# Online Resource 7 Chlorophyll content as a function of Julian days for 199 wheat varieties grown in 2016 and 2017 with no-stress (blue) and stress (red) post-flowering temperature treatments. Raw data were used to build the boxplot.

# Online Resource 8 Marey map of chromosome 4B and physical positions of gene *Rht-B1* (red vertical line) and the QTL for TKW heat stress tolerance (blue vertical line). Physical positions are the position on Chinese Spring refSeq1.0 (IWGSC et al 2018) and genetic positions come from a consensus map between the Renan/Recital genetic map (Rimbert et al. 2018) and the Limagrain genetic map (unpublished data).
